# Advances in Design and Preparation of Biodegradable Inferior Vena Cava Filters

**DOI:** 10.3390/ma19204282

**Published:** 2026-10-10

**Authors:** Jiafan Shen, Hailei Li, Lin Ye

**Affiliations:** 1 School of Materials Science and Engineering, Beijing Institute of Technology, Beijing 100081, China; 3120251113@bit.edu.cn; 2 Department of Surgery, The University of Hong Kong, Hong Kong 999077, China

**Keywords:** biodegradable inferior vena cava filters, biodegradable polymers, degradation kinetics, structural design

## Abstract

Venous thromboembolism (VTE) remains a leading cause of morbidity and mortality worldwide, with pulmonary embolism (PE) constituting its most fatal clinical consequence. Inferior vena cava filters (IVCFs) have been employed as a mechanical prophylactic strategy for patients with contraindications to anticoagulation or recurrent embolic events. However, permanent implantation and limited retrieval success are associated with device-related complications, including caval perforation, migration, fracture, and filter-induced thrombosis. Biodegradable IVCFs have been investigated as an alternative approach to provide temporary protection while avoiding the complications associated with long-term implantation. This review discusses recent advances in biodegradable IVCFs from a material perspective, focusing on material selection, structural design, degradation characteristics, and biological responses. Biodegradable polymers, degradable metallic materials, and shape-memory systems are evaluated regarding their mechanical stability, degradation behavior, vascular compatibility, and functional requirements. Strategies for improving radiopacity while preserving biodegradability are also summarized. Despite ongoing progress, key challenges remain in precisely controlling degradation kinetics, maintaining adequate mechanical support during the therapeutic period, and validating long-term safety through clinical studies. Further efforts in material optimization, biofunctional modification, and individualized fabrication may facilitate the future development of biodegradable IVCFs.

## 1. Introduction

Venous thromboembolism (VTE) is a major vascular disorder primarily manifested as deep vein thrombosis (DVT) and pulmonary embolism (PE) [1]. It has emerged as the third leading cause of cardiovascular mortality worldwide, following acute coronary syndromes and stroke, thereby imposing a substantial global public health burden. According to epidemiological data, the annual incidence of VTE in the general population is approximately 1–2 per 1000 individuals [2], with markedly increased prevalence among elderly patients and those with malignancy, severe trauma, or major surgical interventions. Once DVT develops, thrombotic fragments may detach and migrate through the venous circulation to the right heart system and subsequently into the pulmonary arterial tree under hemodynamic forces, leading to acute PE [3]. Acute high-risk PE can rapidly increase right ventricular afterload, leading to acute right ventricular failure, hemodynamic collapse, and potentially fatal outcomes, and is associated with substantial early mortality [4]. Therefore, effective prevention and clinical management of PE represent a critical priority in the field of cardiovascular medicine.

The inferior vena cava filter (IVCF) is essentially an intravascular implant that relies on the synergistic interplay between material properties and structural design [5]. After implantation, the device is required to function reliably within a dynamic hemodynamic environment characterized by continuous pulsatile flow and complex shear stress. Its functional objectives extend beyond efficient capture of migrating thrombi within the confined lumen of the inferior vena cava (IVC) and also include preservation of venous patency and accommodation to cyclic mechanical loading induced by respiration and postural changes [6]. In this context, minimizing chronic irritation to the venous wall and reducing secondary thrombosis and related complications constitute critical criteria for evaluating filter performance. Since the mid-twentieth century, when stainless steel was first introduced for permanent IVCFs, advances in alloy design and processing technologies have led to the incorporation of cobalt-chromium alloys, titanium alloys, and nickel-titanium alloys (nitinol) into filter fabrication [7]. Through modulation of alloy composition, phase structure, and microstructural features, these materials exhibit improved radial support capability, structural flexibility, and fatigue resistance. With the progressive evolution of these material systems, clinical application of IVCFs has expanded from permanent implantation to more diverse indications. The devices are now widely used in the prevention and management of VTE, especially in clinical settings with a high risk of developing DVT, such as trauma care [8], perioperative management in orthopaedics [9], and the prevention of malignancy-associated thrombosis, thereby establishing an important adjunctive role in the prevention of fatal PE in patients with contraindications to anticoagulation or failure of anticoagulant therapy.

## 2. Non-Degradable IVCFs and Their Limitations

The technological evolution of IVCFs reflects the continuous effort in clinical medicine to maximize protection against PE while minimizing device-related complications. Since the introduction of the Mobin-Uddin umbrella filter [10] in the 1960s, the design of IVCFs has progressed from bulky devices requiring relatively invasive implantation toward minimally invasive and structurally refined systems. Early umbrella-type filters relied on a membrane-supported configuration for thrombus interception, whereas subsequent IVCFs evolved toward open strut-based geometries, including conical and biconical designs [11]. Conical filters use struts converging toward a central apex, whereas biconical filters incorporate opposing conical elements [12]. The conical configuration subsequently became predominant, as its geometry promotes central thrombus accumulation while preserving pathways for caval blood flow around the trapped thrombus [13]. During the 1980s and 1990s, advances in materials science facilitated the maturation of permanent filter designs, particularly with the adoption of nitinol [14]. Owing to their super elasticity and shape memory effect, nitinol enabled filters to be delivered through small-caliber catheters in a highly compact configuration and to self-expand into a predetermined conical architecture upon deployment [15]. This transformation markedly reduced procedural trauma through optimized anchoring mechanisms and refined strut configurations and substantially improved thrombus capture efficiency and implantation stability. Despite these advances, long-term indwelling permanent filters are associated with a spectrum of complications, including IVC perforation, filter tilt or migration, fracture of supporting struts, filter-related thrombosis, and even severe IVC occlusion syndrome [16,17,18]. These adverse events may not only compromise the protective efficacy of the device but also introduce additional clinical risks.

At the beginning of the 21st century, retrievable IVCFs were introduced [19]. These devices incorporate structural elements such as retrieval hooks or retrieval loops that enable the filter to be captured by a snare device and removed under percutaneous interventional procedures. This design allows the filter to be extracted after the period of high thromboembolic risk, thereby reducing long-term complications associated with permanent implantation. This strategy maintains the preventive effect against PE while reducing long-term risks associated with permanent metallic implants, including vascular injury, structural fatigue, and secondary filter-related thrombosis.

Although retrievable filters offer clear conceptual advantages, their application remains challenged by multiple limitations. From a structural mechanics perspective, exposure to pulsatile blood flow and thrombus loading can induce filter tilt or eccentric apposition to the caval wall. Such deformation may displace the retrieval hook or result in endothelial overgrowth covering the hook apex, thereby markedly increasing the technical difficulty of retrieval. In some cases, strut penetration through the IVC wall has been observed during indwelling, which further compromises the feasibility and safety of subsequent removal [20,21]. If retrieval is indicated, current retrievable filters are recommended to be removed within a certain period of time. At the materials level, most currently available retrievable filters are fabricated from nitinol. While this material provides excellent super elasticity and shape memory behavior that are well suited for catheter-based delivery and in situ self-expansion, its pronounced biological inertness implies that, in the event of retrieval failure, the device will remain permanently implanted and continue to expose the patient to long-term device-related complications [22,23].

Collectively, the available evidence indicates that both conventional permanent and retrievable IVCFs are associated with inherent and unavoidable limitations. On the one hand, the long-term complication profile of permanent filters has been well documented. On the other hand, the low retrieval rates observed in clinical practice, rarely exceeding 50% [24,25], substantially compromise the anticipated long-term safety advantages of retrievable devices. Consequently, the current clinical application of IVCFs is characterized by a clear tension between well-defined short-term therapeutic benefits and non-negligible long-term risks. Therefore, there remains an urgent clinical need for key improvements in retrievable filters from the perspectives of material preparation and structural design, so as to achieve better performance in avoiding long-term risks [26,27,28,29].

**Figure 1:**
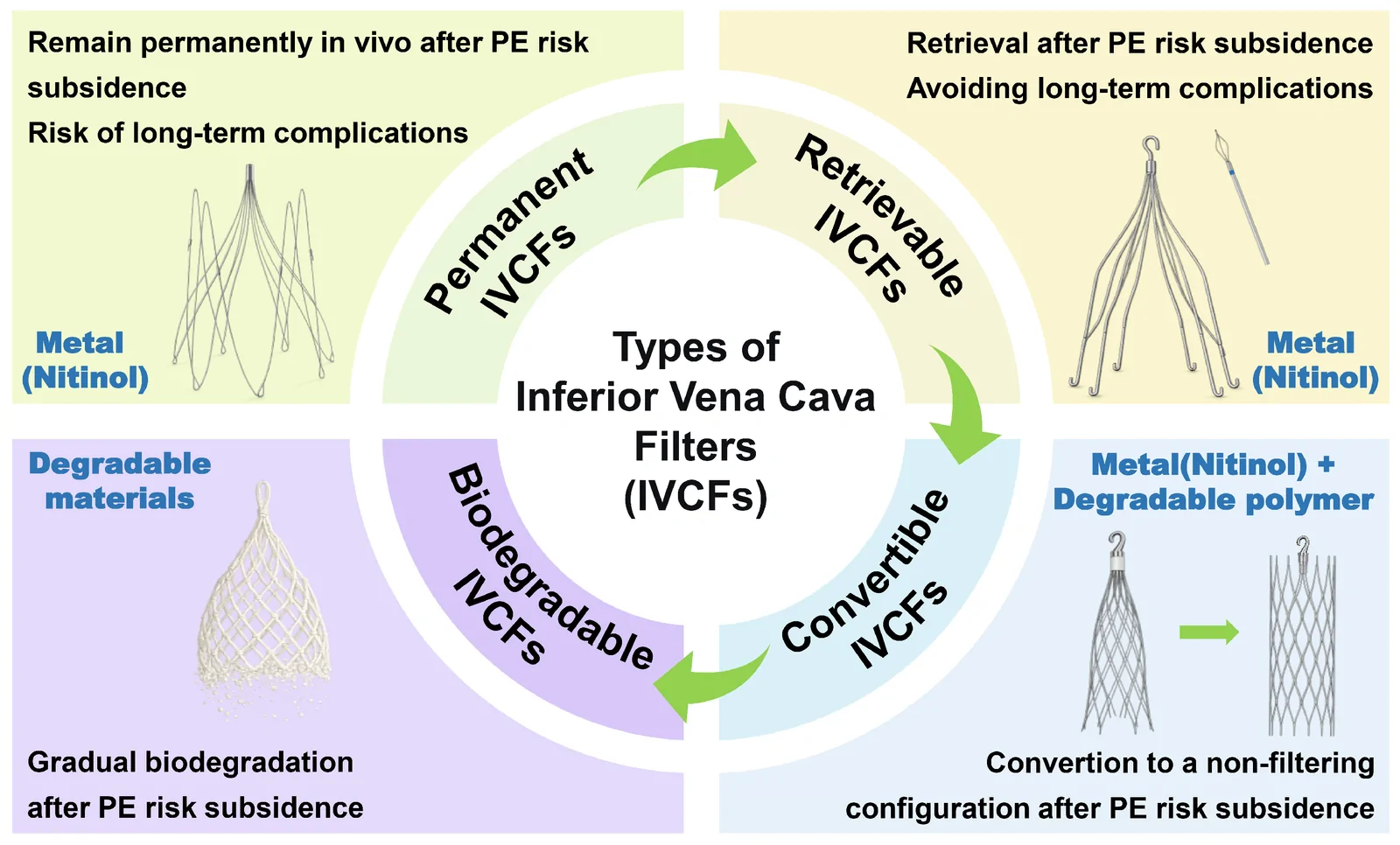
Overview of the four main types of IVCFs, detailing their material compositions, mechanisms, and management strategies following PE risk subsidence.

Recently, two modification strategies for retrievable filters have garnered growing interest. One has focused on surface functionalization with anticoagulant or anti-neointimal hyperplasia coatings to improve long-term patency and retrieval outcomes [30,31]. Antithrombotic agents can prevent in-stent thrombosis, whereas anti-proliferative agents inhibit tissue overgrowth around the filter, thus facilitating the retrieval of retrievable filters. For example, poly(ε-caprolactone) (PCL) polymer and hydrogel coatings were employed for the controlled delivery of antithrombotic and anti-proliferative drugs, respectively. The efficacy in enhancing the retrieval rate of retrievable filters was demonstrated in a sheep vena cava model. Alternatively, another strategy is to adopt biodegradable filters in place of retrievable filters. Biodegradable IVCFs are designed to provide short-term therapeutic benefits during the high-risk period and subsequently degrade in a controlled manner, thereby eliminating the need for secondary retrieval procedures. By virtue of its biodegradable nature, the biodegradable filter effectively eliminates the risks of long-term indwelling in the body. Thus, the development of biodegradable IVCFs that achieve an appropriate balance among biocompatibility, mechanical performance, controlled degradation behavior, and radiopacity has emerged as a research focus at the intersection of biomedical engineering and vascular surgery. Figure 1 outlines the four major categories of IVCFs, comparing their material properties, structural mechanisms, and clinical outcomes following the subsidence of PE risk.

## 3. Materials of Biodegradable IVCFs

The performance of biodegradable IVCFs is fundamentally governed by two interrelated design requirements. First, the filter must possess reliable and sustained thrombus-capturing capability, which relies on maintaining an optimized three-dimensional mesh architecture and adequate radial support during the early post-implantation stage to ensure intimate apposition to the vessel wall and efficient interception of circulating emboli. More importantly, the degradation behavior must be precisely synchronized with the evolution of the mechanical properties. The filter should retain sufficient radial strength and structural integrity throughout the intended period of thromboembolic protection, during which sustained thrombus-capturing capability is required. Subsequently, as the risk of PE declines, the device should undergo controlled biodegradation at a predetermined rate, accompanied by a gradual reduction in mechanical support, while producing biocompatible degradation products that can be safely metabolized or eliminated without leaving permanent foreign materials in vivo.

### 3.1. Biodegradable Polymers

Polymer-based IVCFs offer a versatile platform for coordinating temporary mechanical support with programmed biodegradation [32,33]. A variety of biodegradable and biocompatible polymers, including aliphatic polyesters, polyanhydrides, polyorthoesters, polycarbonates, and polyurethanes, have been explored for vascular implants, among which aliphatic polyesters remain the most representative because their hydrolytic degradation, mechanical properties, and resorption profiles can be modulated over a broad range [34,35,36,37]. Compared with metals, biodegradable polymers generally exhibit elastic moduli closer to those of compliant venous tissues, which may reduce mechanical mismatch at the implant–vessel interface and thereby limit sustained mechanical loading of the vessel wall [38,39]. For biodegradable polymer filters, mechanical performance is inherently coupled to hydrolytic degradation, as progressive chain scission can reduce molecular weight, alter polymer morphology, and ultimately compromise load-bearing capacity. The rate and extent of this mechanical decay depend on polymer chemistry, molecular weight, molecular-weight distribution, crystallinity, and processing history [40,41]. Among commonly investigated aliphatic polyesters, poly(glycolic acid) (PGA), poly(lactic acid) (PLA), and PCL exhibit distinct degradation kinetics because of differences in backbone structure, hydrophilicity, stereochemistry, and crystalline morphology [42]. PGA generally undergoes relatively rapid hydrolytic degradation, reflecting the combined effects of its relatively hydrophilic backbone, water uptake, and polymer morphology [43]. PLA exhibits intermediate degradation kinetics, but its behavior varies substantially with stereochemical composition. PLA encompasses stereoregular PLLA and PDLA, which are capable of forming ordered crystalline domains, as well as stereoirregular poly(D,L-lactic acid) (PDLLA), which is typically amorphous. Under comparable molecular weight and processing conditions, PLLA generally degrades more slowly than PDLLA because crystalline regions restrict water penetration and reduce the accessibility of ester bonds to hydrolysis [44,45,46]. Importantly, crystallinity should not be treated as an intrinsic and invariant property of these polymers. In PLA-based materials, the extent of crystallization depends on stereoregularity, molecular weight, cooling conditions, thermal history, and post-processing treatments such as annealing; consequently, PLLA can range from nearly amorphous to highly crystalline states, with crystallinity exceeding 50% under suitable thermal treatments and with corresponding changes in hydrolytic degradation and mechanical properties [47,48]. PCL generally undergoes slower hydrolytic degradation and can retain its mechanical integrity for longer periods than PGA and PLA under comparable conditions, owing primarily to its hydrophobic methylene-rich backbone and relatively low hydrolytic susceptibility, while its crystalline domains can further restrict water transport and chain cleavage [49,50,51]. Nevertheless, the crystallinity and degradation kinetics of PCL are also sensitive to molecular characteristics and processing history. Beyond homopolymer composition, copolymerization provides an additional means of regulating degradation by altering chain regularity and crystallization behavior, thereby modifying the relative fractions and spatial organization of crystalline and amorphous domains [52,53]. Molecular weight provides a further determinant of mechanical persistence. Higher initial molecular weight generally provides greater chain entanglement and load-bearing capacity during the early stages of degradation, whereas substantial mechanical deterioration often occurs after sufficient chain scission has reduced the molecular weight and disrupted the load-bearing polymer network [54,55,56,57]. Accordingly, PGA generally exhibits more rapid hydrolytic degradation than PLA and PCL under comparable conditions, whereas PCL typically exhibits comparatively high resistance to hydrolytic chain scission. This general trend should not be regarded as an absolute ranking, because the functional lifetime of a biodegradable filter is governed by the combined effects of polymer chemistry, stereochemistry, molecular weight, crystallinity, morphology, and processing history. Rational control over these parameters therefore provides a means of tailoring the duration of mechanical support to the intended protection window of biodegradable IVCFs while minimizing premature loss of structural integrity or prolonged material persistence.

**Figure 2:**
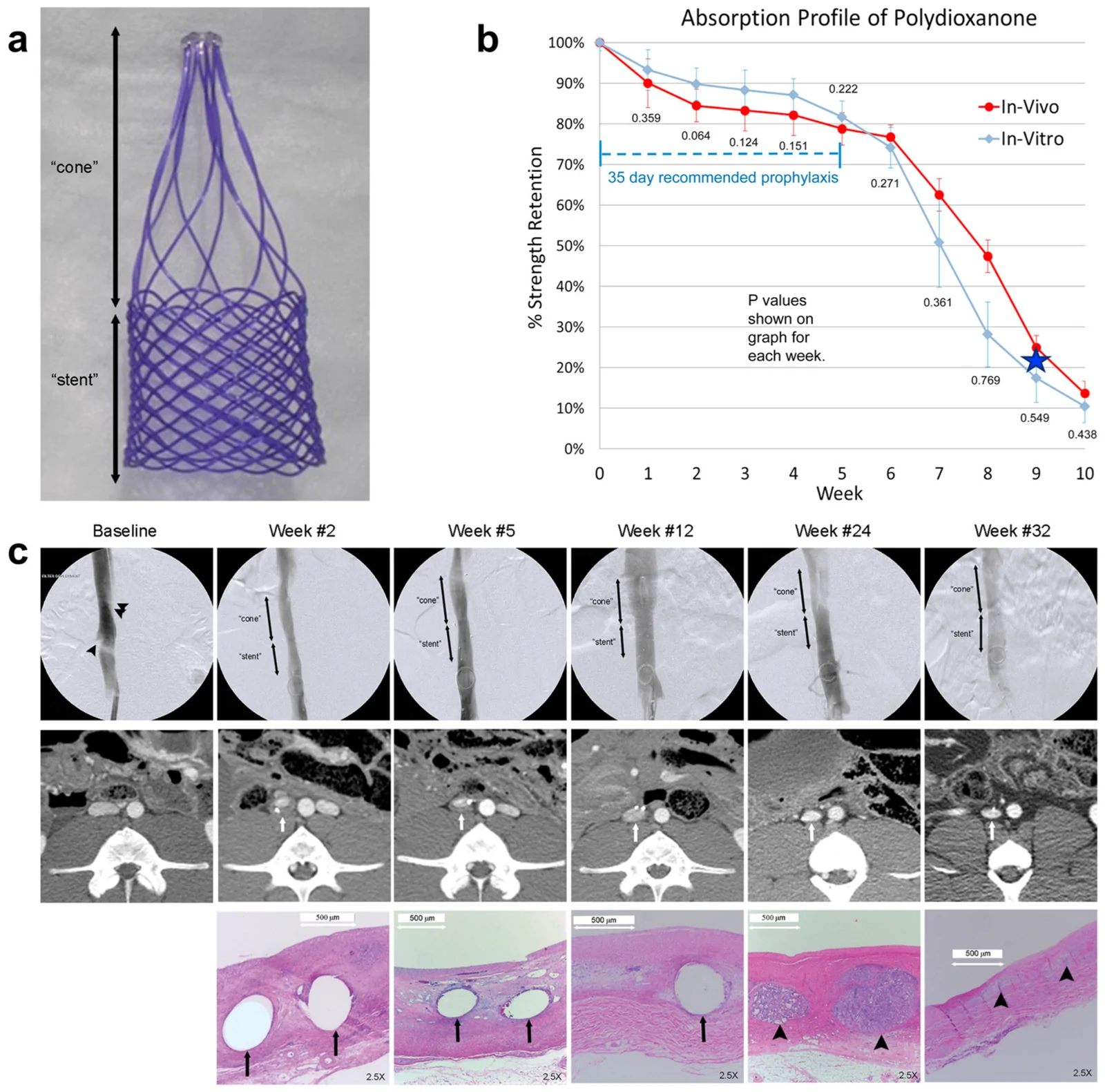
Design, degradation behavior, and in vivo performance of a PPDO-based biodegradable IVCF. (**a**) Photograph of the PPDO-based biodegradable IVCF. (**b**) Tensile strength retention of PPDO during in vitro and in vivo degradation over time. The blue star denotes a PPDO suture segment retrieved from the capture basket of a biodegradable IVCF after 9 weeks of implantation, including 3 weeks of thrombus exposure. (**c**) Representative fluoroscopic, axial Computed Tomography (CT), and hematoxylin–eosin (H&E) staining images showing temporal changes in the IVC after infrarenal implantation of the IVCF. In the top row, double-headed arrows delineate the cone and stent components of the filter, and white arrows in the middle row indicate the stent component. Black arrows in the bottom row indicate embedded PPDO fragments within the caval wall. Early caval wall hyperplasia was most pronounced at weeks 2 and 5, followed by progressive degradation of the PPDO fragments and attenuation of the associated foreign-body granulomatous inflammation by week 32. Reproduced from Refs. [59,60] with permission.

Poly(p-dioxanone) (PPDO) is the most extensively investigated biodegradable polymer for IVCFs because it combines substantial short-term mechanical strength retention with progressive hydrolytic degradation and subsequent resorption [58]. This profile allows PPDO-based filters to maintain structural integrity during the initial period of thromboembolic protection while avoiding indefinite material persistence. Eggers et al. [59,60]. subsequently established the feasibility of PPDO-based IVCFs through a series of sequential in vitro, animal, and clinical studies, providing evidence for their mechanical performance, thrombus-capturing capability, degradation behavior, and long-term safety. As shown in Figure 2a, the filter was fabricated almost entirely from medical-grade PPDO monofilaments, with only a small metallic marker incorporated for radiographic localization. The device consisted of a stent segment for fixation to the caval wall and a conical filtering segment.

The principal objective of these studies was to determine whether the degradation behavior of PPDO could maintain sufficient mechanical integrity throughout the transient therapeutic window required for PE prevention. Both an in vitro circulating flow model and a porcine implantation model demonstrated highly consistent degradation profiles. Throughout the clinically relevant 35-day protection period, PPDO retained 82% of its initial tensile strength in vitro, compared with 79% in vivo, demonstrating that the in vitro degradation model closely paralleled the in vivo strength attenuation profile. Thereafter, the material underwent accelerated mechanical deterioration, with tensile strength declining to approximately 20% of its initial value by Week 9, as depicted in Figure 2b. These findings indicate that the PPDO filter was intentionally designed to preserve sufficient structural integrity during the therapeutic window rather than maintain unnecessary long-term mechanical support, thereby achieving temporal synchronization between mechanical degradation and clinical demand.

The influence of degradation on filtration performance was further evaluated by introducing thrombi at different implantation stages. Regardless of whether thrombi were released immediately after implantation or at Days 14 and 35, the experimentally introduced thrombi were successfully captured and retained until autologous thrombolysis. These findings demonstrate that progressive degradation of PPDO did not impair thrombus-capturing capability during the clinically relevant protection period and that the filter maintained sufficient mechanical integrity beyond the target implantation duration.

To further evaluate the long-term degradation behavior and biosafety of PPDO, a subsequent in vivo study was conducted in 11 swine with follow-up extending to 32 weeks. No evidence of device migration, caval penetration, IVC thrombosis, or PE was observed during the study period. Figure 2c shows neointimal formation around the filter, which was observed as early as 2 weeks after implantation and accompanied by fibromuscular proliferation during the subsequent follow-up period. At 32 weeks, residual PPDO fragments were identified within the caval wall and associated with foreign-body granulomatous reactions. Small foci of PPDO fragments were occasionally detected in pulmonary tissue at late time points, and these fragments were associated with macrophage infiltration and granulomatous inflammation. Serial laboratory evaluations revealed no significant deviations from baseline hematologic, hepatic, renal, or coagulation parameters, supporting the favorable systemic safety profile of PPDO degradation.

The subsequent randomized controlled study further strengthened the preclinical evidence by directly comparing the absorbable PPDO filter with a benchmark metallic filter in a Good Laboratory Practice porcine model. Among 24 swine, 16 received the absorbable filter and 8 received the metallic control, with follow-up to 32 weeks. Compared with the metallic filter, the PPDO filter showed lower incidences of caval wall perforation and thrombosis, while no PE was detected in either group during follow-up. By 32 weeks, the polymer was completely to nearly completely resorbed, and the treated IVC was macroscopically comparable to adjacent normal veins apart from the radiopaque markers, providing comparative evidence for the long-term safety and vascular restoration associated with an absorbable filter [61]. These findings subsequently supported translation into a prospective first-in-human study of eight patients. Seven filters were implanted prophylactically before orthopedic or gynecologic surgery and one was implanted for DVT, with imaging follow-up through 36 weeks. No symptomatic or imaging-detected PE was observed at the planned 5-week assessment, and no filter migration, embolization, caval perforation, or stenosis was identified during follow-up. The study achieved 100% clinical success and 89% technical success, providing initial clinical feasibility and short-term safety evidence for transient PE protection without subsequent filter retrieval [62].

Collectively, these studies demonstrate that the clinical feasibility of PPDO-based IVCFs arises from the close coordination between degradation kinetics and mechanical retention rather than prolonged structural persistence. By maintaining sufficient mechanical integrity and thrombus-capturing capability throughout the period of highest PE risk, followed by progressive loss of mechanical function and gradual resorption thereafter, PPDO establishes a material design paradigm in which degradation behavior is matched to the temporal requirements of thromboembolic protection. This degradation–mechanical coupling has subsequently become the fundamental design principle for the development of fully biodegradable polymeric IVCFs.

**Figure 3:**
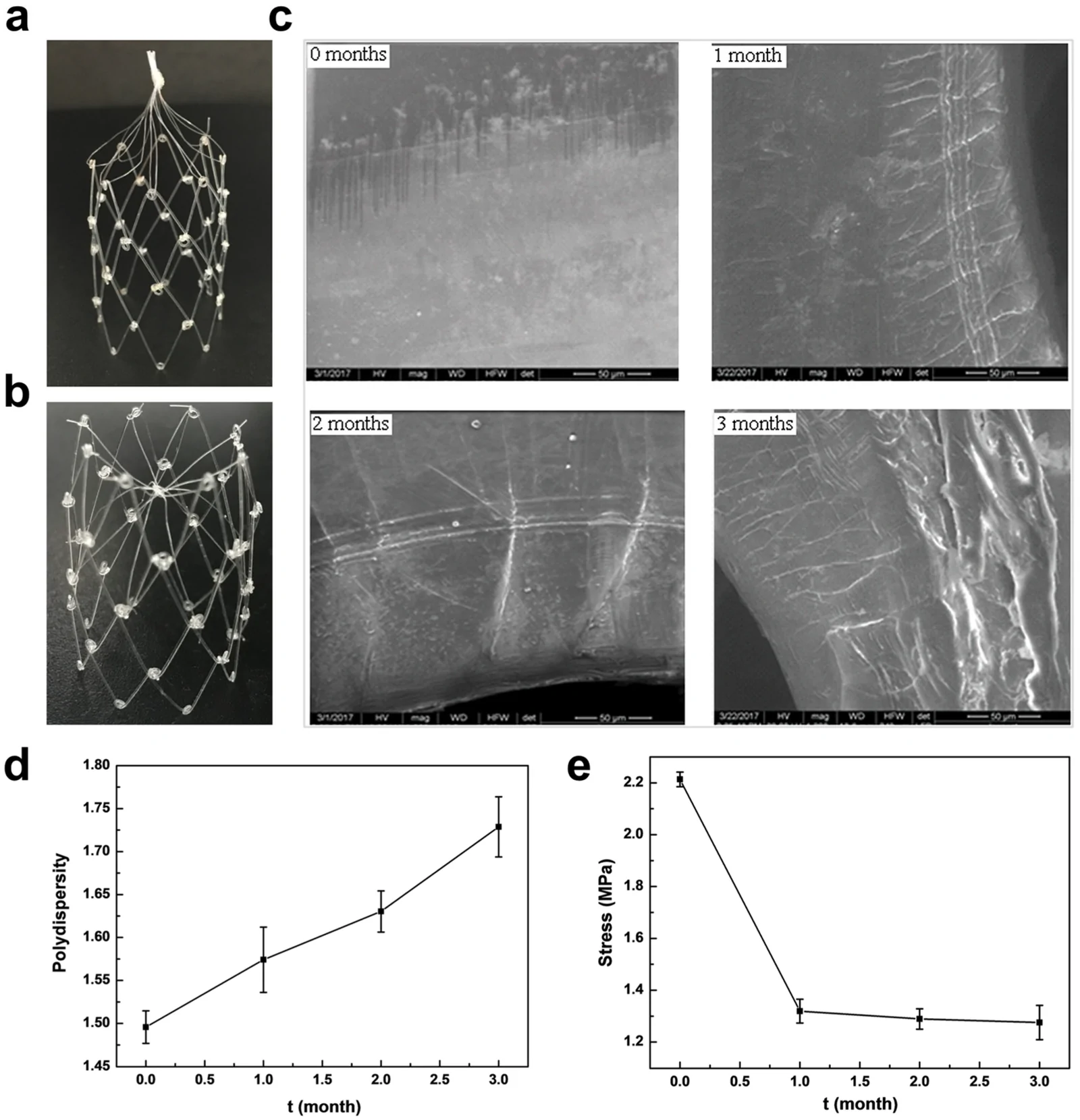
Design and degradation behavior of a PLA-based biodegradable IVCF. (**a**,**b**) Filtration structures with single-layer and double-layer connections. (**c**) SEM images of the filter surface after 0, 1, 2, and 3 months of degradation (scale bar = 50 μm). (**d**,**e**) Polydispersity and radial support strength as a function of degradation time. Reproduced from Ref. [63] with permission.

PLA has also been extensively investigated as a candidate material for biodegradable IVCFs because of its relatively high elastic modulus, favorable biocompatibility, and established clinical use in biodegradable medical devices. Unlike metallic filters, however, the long-term performance of PLA-based IVCFs is governed not only by their initial mechanical properties but also by the ability to preserve sufficient structural integrity throughout polymer degradation. Cui et al. [63]. developed a biodegradable IVCF braided entirely from PLA filaments and systematically evaluated the relationship among polymer degradation, mechanical retention, and thrombus-capturing performance. Figure 3a,b illustrate the two connection strategies between the PLA-wire filtration mesh and the support frame: a single-layer arrangement forming a conical filter, and a double-layer design engineered to boost radial fixation force and optimize clot trapping. In vitro degradation studies demonstrated that hydrolysis induced progressive molecular chain scission, with the number-average molecular weight decreasing from 133,747 to 44,196 after 16 weeks of degradation. Scanning electron microscopy (SEM) analysis in Figure 3c reveals a gradual transition from a smooth fiber surface to one characterized by surface cracking and erosion, indicating that molecular degradation progressively evolved into macroscopic structural deterioration. Figure 3d illustrates that polydispersity elevated monotonically with prolonged degradation time. These observations demonstrate that degradation of PLA is initiated at the molecular level before becoming manifested as structural damage capable of compromising device performance.

The degradation-induced evolution of polymer structure was closely associated with mechanical deterioration of the filter. The initial radial support strength of 2.21 MPa decreased to 1.32 MPa after four weeks of degradation and subsequently declined at a slower rate as depicted in Figure 3d. Notably, the reduction in radial support closely paralleled the decrease in molecular weight, indicating that mechanical deterioration was primarily governed by polymer chain scission rather than geometric instability of the filter architecture. These findings suggest that molecular weight retention represents a key determinant of the effective service life of fully biodegradable polymeric IVCFs and that degradation kinetics directly dictate the duration over which sufficient mechanical support can be maintained.

Because degradation inevitably reduces mechanical stability, Cui et al. further investigated whether structural optimization could compensate for the intrinsic mechanical limitations of biodegradable polymers. Using a conventional single-layer configuration, the filter achieved a thrombus capture efficiency of 70% in an in vitro circulation model. Following introduction of an additional top connection to establish a double-layer supporting architecture, the capture efficiency increased to 90%. This improvement demonstrates that filter performance depends not only on material properties but also on structural design. Appropriate topological optimization can effectively enhance structural stability during degradation, thereby partially offsetting the relatively low stiffness of biodegradable polymers while improving thrombus interception efficiency.

**Figure 4:**
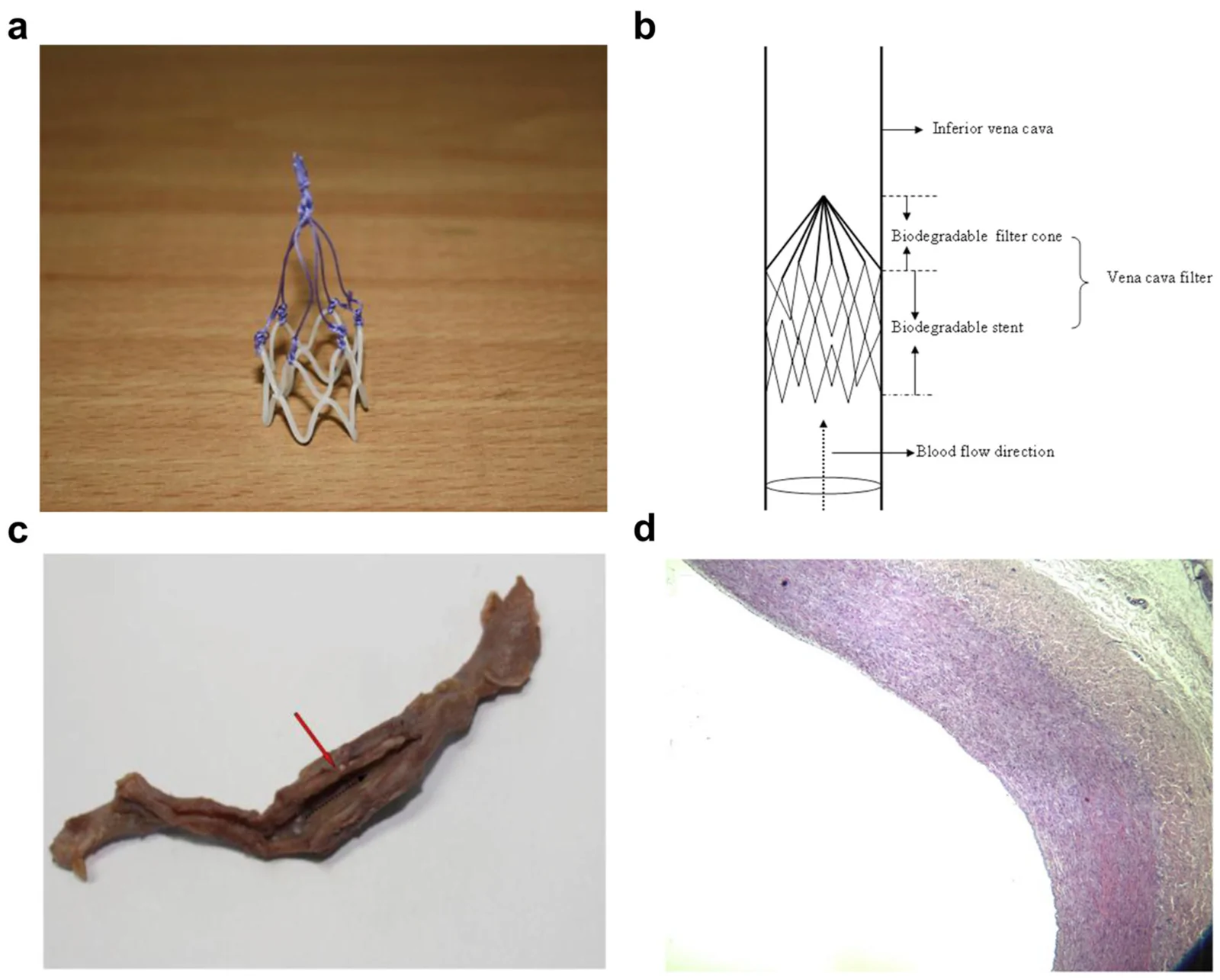
(**a**) Biodegradable vena cava filter used for implantation in a canine IVC model. (**b**) Schematic drawing of the biodegradable vena cava filter. (**c**) Opened IVC specimen showing embedding of the biodegradable stent within the caval wall; the red arrow indicates the direction of blood flow. Vessel wall thickening and inflammatory responses were observed after implantation. (**d**) H&E staining of the IVC wall showing intimal and medial thickening, unclear separation between the intima and media, and smooth muscle cell hyperplasia with inflammatory cell infiltration in the tunica media (40× magnification). Reproduced from Ref. [64] with permission.

Zhang et al. [64]. prepared a biodegradable filter consisted of two parts, a filter cone and a stent. As shown in Figure 4a,b, the filter cone was constructed of six PGA polymer strands anchored to a handmade absorbable stent. Central IVC fixation was accomplished by the absorbable stent, which was made of PCL. Device insertion was performed through a 9F sheath under ultrasound guidance on 10 adult beagles.

The degradation profile of the filter was designed to match the temporal characteristics of PE risk. Clinical evidence indicates that the incidence of post-traumatic PE peaks within the first 2 weeks after injury. Accordingly, the PGA filtering filaments underwent complete hydrolytic degradation within approximately 6 weeks, whereas the PCL supporting framework exhibited an in vivo degradation period exceeding 24 months. This differential degradation behavior ensured that thrombus-capturing capability was maintained throughout the clinically relevant protection period while preserving anchoring stability after resorption of the filtering basket, thereby reducing the likelihood of premature filter migration associated with rapid degradation of the entire device. The biological performance of the filter was subsequently evaluated in a canine implantation model. Following 6 weeks of implantation, all animals survived without procedure-related mortality or local infection. Gross examination demonstrated complete degradation of the PGA filtering component in 9 of 10 adult beagles weighing 12–15 kg each, whereas the remaining case exhibited residual filter filaments incorporated into the venous wall with localized neointimal coverage, resulting in approximately 80% luminal stenosis. In contrast, the PCL supporting framework remained structurally intact in all animals and became embedded within the caval wall without obvious geometric deformation or clinically significant vena cava narrowing. These findings indicate that differential degradation effectively maintained positional stability during the transition from functional filtration to structural resorption, although incomplete degradation of the filtering component may still compromise local vascular remodeling in isolated cases.

Histopathological evaluation provided further insights into the tissue response associated with the differential degradation strategy, with representative staining images presented in Figure 4b,c. No PE caused by filter dislodgement or degradation fragments was detected during follow-up. However, evident local foreign-body reactions were observed, including inflammatory cell infiltration, intimal and medial thickening, smooth muscle proliferation, and granuloma formation surrounding residual polymer fragments. These responses remained confined to the implantation site, indicating localized tissue remodeling without apparent systemic toxicity. Collectively, these findings demonstrate that spatial separation of degradation functions provides an effective strategy for balancing thrombus interception, structural stability and biological safety in fully biodegradable polymeric IVCFs.

Overall, PPDO currently has the strongest translational evidence, whereas PLA and PGA/PCL require more comprehensive in vivo validation before their proposed mechanical and degradation advantages can be considered clinically meaningful. In the future, on the one hand, more animal experiments and clinical trials should be promoted to verify whether these material systems can truly be applied to biodegradable IVCFs; on the other hand, the research focus should be shifted from achieving biodegradability to establishing a predictable temporal match among mechanical retention, filtration function, fixation stability, and tissue response.

Representative studies of biodegradable polymeric IVCFs are summarized in Table 1, including material selection, degradation behavior, animal evidence, major findings or limitations and clinical studies. Collectively, these studies highlight that the therapeutic value of a biodegradable IVCF depends not only on its degradation characteristics but also on whether mechanical protection against PE is maintained for the clinically required period. The optimal therapeutic window of an IVCF is indication dependent and should primarily correspond to the period during which mechanical protection against PE remains clinically necessary. For conventional retrievable IVCFs, the FDA recommends considering retrieval once protection from PE is no longer required, with approximately 29–54 days often cited as the period during which the benefit–risk profile may favor removal in patients with transient PE risk [65]. A similar principle may be applied to biodegradable IVCFs, such that their thrombus interception and PE prevention capabilities should be maintained in vivo for at least the duration of this clinically relevant treatment period.

### 3.2. Biodegradable Metals

To date, biodegradable metallic IVCFs have not yet been specifically reported or evaluated, although biodegradable metallic vascular stents have been investigated as temporary vascular implants that provide mechanical support during tissue healing. The rapid development of biodegradable metallic vascular stents and other temporary intravascular implants has provided increasing evidence for the feasibility of using biodegradable metals in vascular devices. Although vascular stents and IVCFs differ in clinical function, both devices require sufficient mechanical integrity throughout a predefined therapeutic period, followed by gradual loss of structural function through controlled degradation. Therefore, the coupling between degradation kinetics and mechanical performance established in biodegradable metallic vascular implants provides an important materials and design basis for the future development of biodegradable metallic IVCFs. In this context, biodegradable metals are considered as a prospective platform that may provide greater mechanical strength, stiffness, and structural support than biodegradable polymers.

However, unlike vascular scaffolds, IVCFs are exposed to continuous venous blood flow, cyclic bending and repetitive thrombus loading rather than sustained radial compression from the vessel wall. Consequently, the key performance requirement shifts from maintaining vascular patency to preserving structural stability and thrombus capture efficiency throughout degradation. This distinction implies that, in addition to degradation kinetics and initial mechanical strength, fatigue resistance and degradation-dependent retention of filtration performance should become primary evaluation criteria for biodegradable metallic IVCFs. Future investigations should establish quantitative relationships among degradation kinetics, mechanical evolution, fatigue durability and thrombus capture performance under physiological venous conditions to define material design criteria for biodegradable metallic IVCFs.

### 3.3. Shape Memory Materials

Compared with biodegradable polymers and biodegradable metals, shape memory materials offer a distinct strategy for biodegradable IVCFs by addressing a key challenge that neither class fully resolves, namely the integration of catheter-based delivery, reliable in situ deployment, and temporary mechanical support [66,67,68]. Biodegradable polymers provide favorable biocompatibility and tunable degradation but are often limited by insufficient radial strength, whereas biodegradable metals offer superior load bearing capacity but lack the deformability required for compact catheter loading and controlled post implantation expansion. Shape memory polymers help bridge this gap by combining large reversible deformation with programmed shape recovery, thereby enabling minimally invasive delivery together with structural recovery after implantation [69,70]. Their clinical applicability still depends on balancing shape recovery, mechanical stability, and degradation behavior, because premature loss of integrity compromises thrombus capture, whereas excessive persistence diminishes the benefit of biodegradation [71].

**Figure 5:**
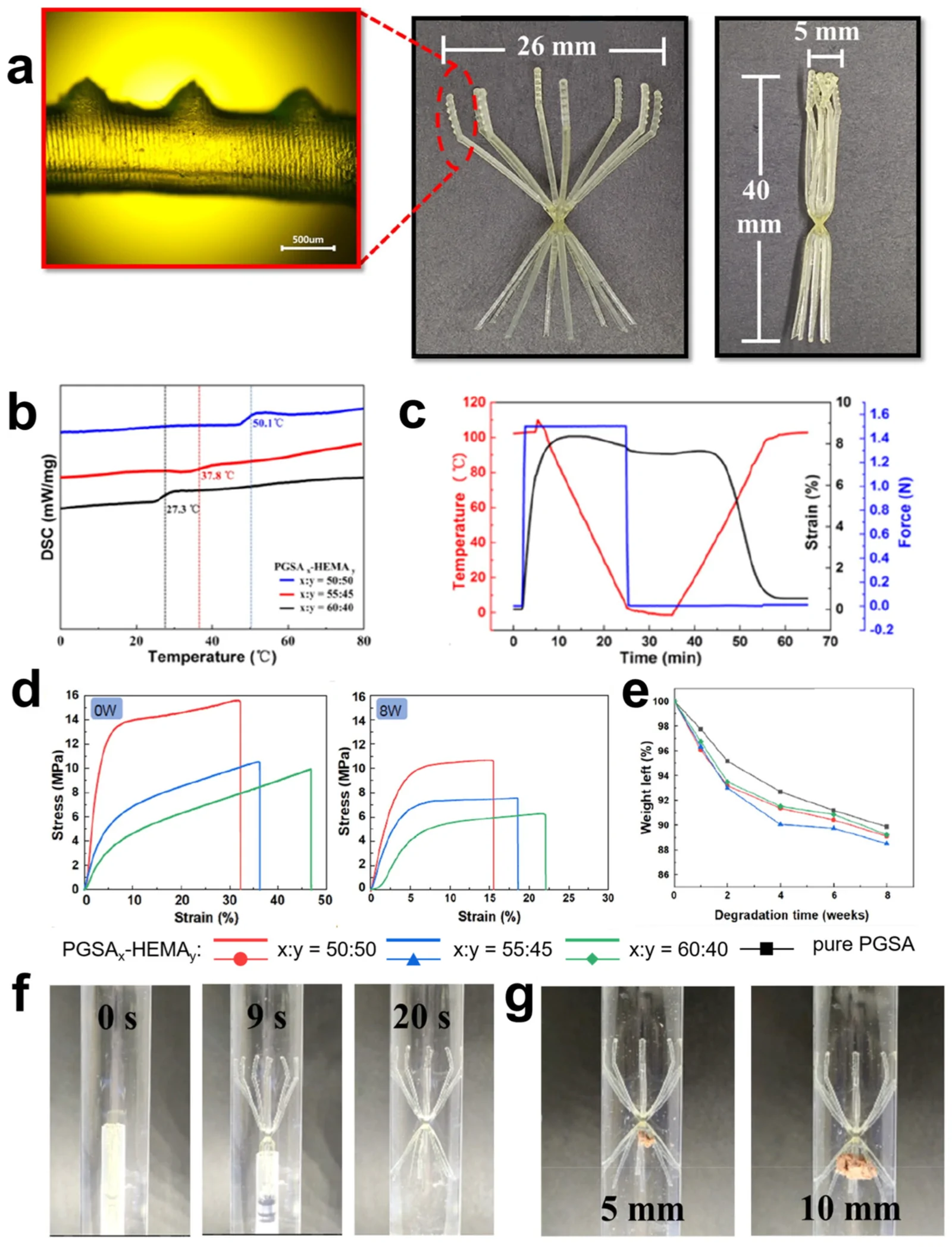
Design and functional characterization of a shape-memory biodegradable IVCF. (**a**) Photographs of the printed IVCF before and after deformation. The inset shows an optical microscopy image of the tip of an upright filter strut. (**b**) Differential scanning calorimetry (DSC) curves of PGSA-PHEMA. (**c**) Shape memory behavior of PGSA_55_-HEMA_45_ evaluated by cyclic thermomechanical testing. (**d**) Stress–strain curves of photocured PGSA-PHEMA after 0 and 8 weeks of in vitro degradation. (**e**) Mass loss profiles of photocured PGSA and PGSA-PHEMA during in vitro degradation. (**f**) Sequential photographs showing in vitro deployment of the IVCF. (**g**) Representative photographs showing in vitro thrombus capture by the IVCF. Adapted from Ref. [72] with permission.

As shown in Figure 5a, Qu et al. [72]. developed a four-dimensional (4D) printed IVCF based on a photocrosslinked copolymer poly(glycerol sebacate) acrylate-co-hydroxyethyl methacrylate (PGSA-co-HEMA). The transition temperature was tuned to 37.8 °C, enabling the compressed filter to recover its predefined geometry immediately after implantation under physiological conditions, as illustrated in Figure 5b. Incorporation of the rigid poly(2-Hydroxyethyl methacrylate) (PHEMA) segment reinforced the elastic poly(glycerol sebacate) acrylate (PGSA) network, increasing tensile strength to approximately 10–16 MPa while maintaining excellent shape memory performance. As shown in Figure 5c, the optimized composite exhibited a shape fixity ratio of 97.3% and a recovery ratio of 92.9%. This structure demonstrates that regulating network composition simultaneously controls thermal activation and mechanical support, both of which are prerequisites for minimally invasive deployment.

The coordination between degradation behavior and mechanical retention is essential for ensuring functional stability, with related evaluations provided in Figure 5d,e. The copolymer exhibited gradual hydrolytic degradation, with only approximately 11% mass loss after 8 weeks. Although degradation progressively reduced tensile properties, the remaining mechanical strength was sufficient to preserve filter function throughout the expected period of PE protection. These findings indicate that an appropriate degradation profile should preserve mechanical integrity during the therapeutic window rather than maximize degradation rate, thereby ensuring functional support before gradual resorption.

The material also demonstrated favorable biological performance. Endothelial cells adhered and proliferated normally on the polymer surface, platelet adhesion remained minimal, and the hemolysis ratio was below 1%, indicating good blood compatibility. Following subcutaneous implantation, only a transient mild inflammatory response was observed, which gradually resolved during the 12-week observation period without obvious pathological abnormalities. These results suggest that the degradation process does not induce persistent adverse tissue responses.

Functional evaluation, presented in Figure 5f,g, further verified the feasibility of this material system. A filter with an expanded diameter of 26 mm was compressed into a 5 mm delivery catheter and recovered within 20 s at physiological temperature. In a simulated venous circulation model, complete capture of both 5 mm and 10 mm thrombus analogues was achieved without filter migration or tilting, demonstrating that shape recovery and retained mechanical stability jointly determine early embolus interception after implantation.

Despite these encouraging results, current evidence remains limited to in vitro degradation, subcutaneous implantation and benchtop flow simulation. The evolution of mechanical properties during degradation under physiological venous loading has not been established, nor has long-term thrombus capture performance been validated in large-animal vena cava models. Future studies should therefore focus on quantitatively correlating degradation kinetics with mechanical reliability and filtration performance to establish rational design criteria for biodegradable shape memory vena cava filters.

Beyond this proof of concept, recent advances in 4D printing suggest that shape-memory behavior can be further coupled with programmed structural transformation and remotely controlled actuation. Choudhury et al. [73]. demonstrated design-encoded dual shape-morphing and shape-memory in 4D-printed PLMC structures, enabling the formation of hollow tubular geometries and subsequent shape recovery at physiological temperature. Although demonstrated in vascular grafts rather than IVCFs, this approach highlights the potential of using printing architecture and material anisotropy to encode complex three-dimensional vascular geometries, rather than relying solely on recovery of a directly printed final shape. More recently, NIR-responsive SMP composites have enabled non-contact shape activation together with fluorescence and photoacoustic imaging in 4D-printed biomedical constructs, suggesting a potential route toward remotely triggered and image-guided deployment [74]. Although the applicability of this strategy to deep intravascular IVCFs remains to be established, such approaches illustrate how future 4D-printed devices may progress from predefined filter geometries toward more programmable endovascular architectures with greater control over shape transformation and deployment.

Despite these promising advances, current evaluations of 4D-printed biodegradable IVCFs remain confined to static in vitro degradation, subcutaneous models, and benchtop flow circuits. The evolution of radial strength and shape recovery during degradation under physiologically relevant venous loading remains insufficiently characterized, and long-term thrombus capture efficacy has not been validated in large-animal vena cava models. Moreover, translating geometry-programmed and remotely activated 4D-printed systems to biodegradable IVCFs will require systematic evaluation of deployment accuracy, structural reliability, stimulus accessibility, and biological safety. Future research should therefore correlate degradation-induced mechanical decay with dynamic hemodynamic and filtration performance, while integrating programmable shape transformation, appropriate stimulus responsiveness, and tailored three-dimensional architectures to establish rational design criteria for next-generation biodegradable endovascular devices.

## 4. Design of Biodegradable IVCFs

### 4.1. Degradation Timing and Functional Matching

Successful biodegradable IVCFs require precise temporal coordination between degradation behavior and evolving functional demands throughout implantation rather than simply maximize material persistence. Clinical evidence indicates that PE can occur very early after major trauma, with a substantial proportion of events detected within the first 3 days after injury [75,76]. Therefore, biodegradable IVCFs should be designed to preserve sufficient radial support, structural integrity, and thrombus capture capability throughout this high-risk period while withstanding continuous venous deformation and hemodynamic loading. Once the period of highest thromboembolic risk has passed, the device should progressively transition from an active filtering state toward a non-filtering configuration while preserving IVC patency and, ideally, undergoing complete and safe resorption. Degradation must be coordinated with the duration of thromboembolic protection. However, achieving this precisely choreographed degradation profile is highly challenging, as it demands stringent control over material chemistry, microstructure, and mechanical properties to simultaneously satisfy conflicting requirements, maintaining robust strength in the short term yet enabling timely and safe resorption thereafter. Such multifaceted and dynamic performance criteria are seldom attainable with conventional biodegradable polymers or metals alone, yet through ingenious structural design, such as hierarchical architectures or spatially graded degradation profiles, it may be possible to reconcile these conflicting demands.

### 4.2. Bioconvertible IVCFs

**Figure 6:**
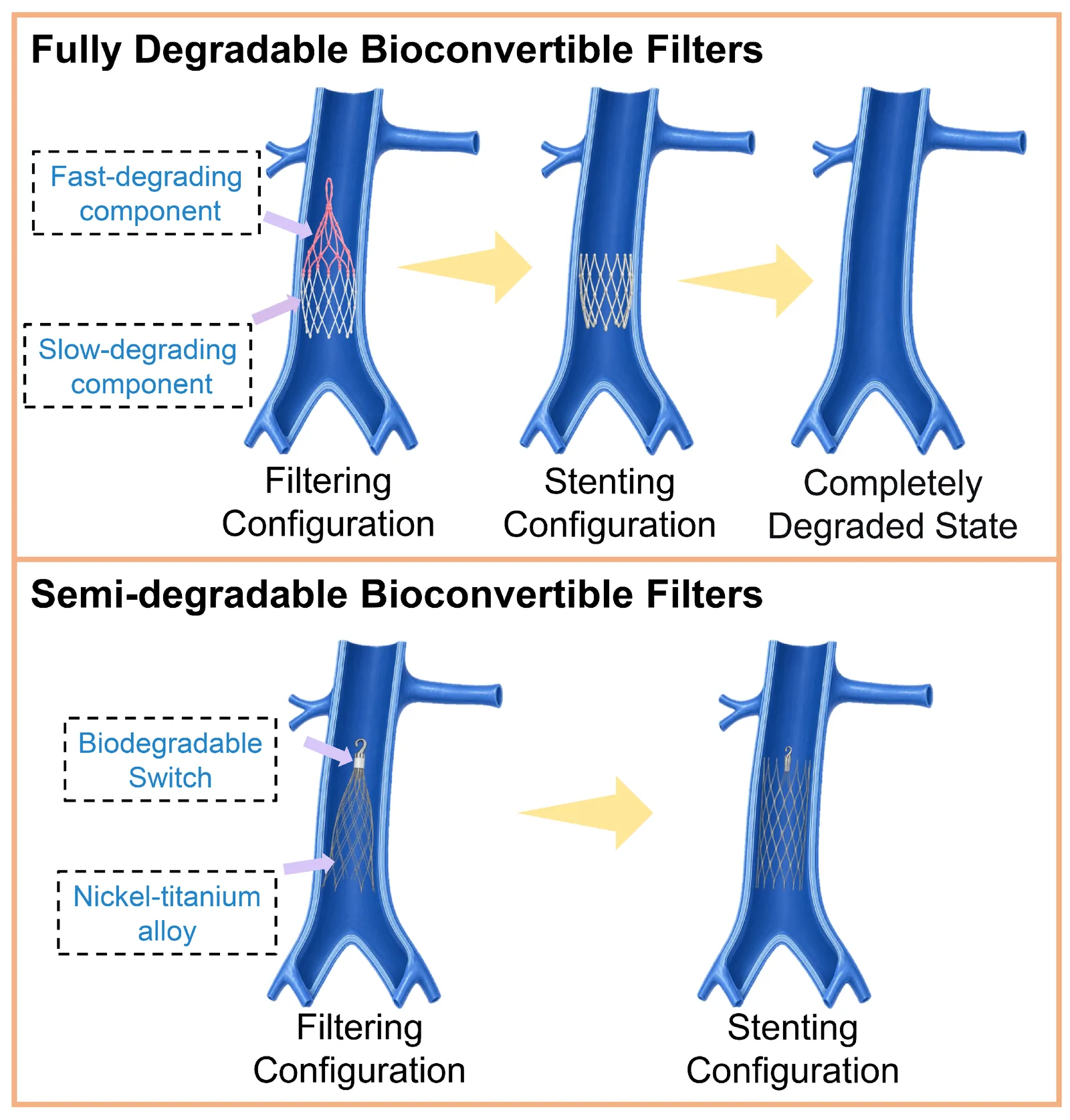
Schematic diagram of fully degradable bioconvertible filters and semi-degradable bioconvertible filters.

The convertible filter is a typical representative that meets the above requirements through structural design. As shown in Figure 6, the convertible filter features a design in which the tip trapping structures are fabricated from rapidly degrading materials, while the main body of the filter is made from slowly degrading materials or conventional non-degradable nitinol. After the short-term therapeutic task is completed, the tip trapping structures degrade rapidly, allowing the filter to transform into a stent-like structure that becomes fully apposed to the vessel wall. This design minimizes the hemodynamic impact of the residual stent-like structure remaining in the lumen, thereby effectively reducing the long-term complications associated with conventional filters left indwelling in the body over extended periods. Therefore, this innovative filter design concept well fulfills the goal of biodegradable filters in balancing short-term therapeutic efficacy with long-term safety.

#### 4.2.1. Fully Degradable Bioconvertible Filters

As discussed above, Zhang and Li [64] proposed a fully biodegradable IVCF based on a differential degradation strategy by integrating two biodegradable polymers with distinct degradation kinetics. The device consisted of a fastly degrading PGA conical filtering basket responsible for thrombus interception and a slowly degrading PCL supporting framework providing anchoring and positional stability. Rather than relying on simultaneous degradation of the entire device, this functional partitioning enabled the filtering component and supporting structure to degrade sequentially according to their respective clinical roles. Although the authors did not explicitly define this device as a bioconvertible filter, it can be conceptually classified as a fully degradable bioconvertible filter based on its sequential functional transformation. Following implantation, the device initially functions as a conventional filter to capture thrombi. As the fastly degrading filtering basket resorbs, the device transitions from a filtering configuration to a stenting configuration while maintaining caval support. Subsequently, the slowly degrading supporting framework gradually degrades, resulting in complete device resorption without any permanent implant remaining.

**Figure 7:**
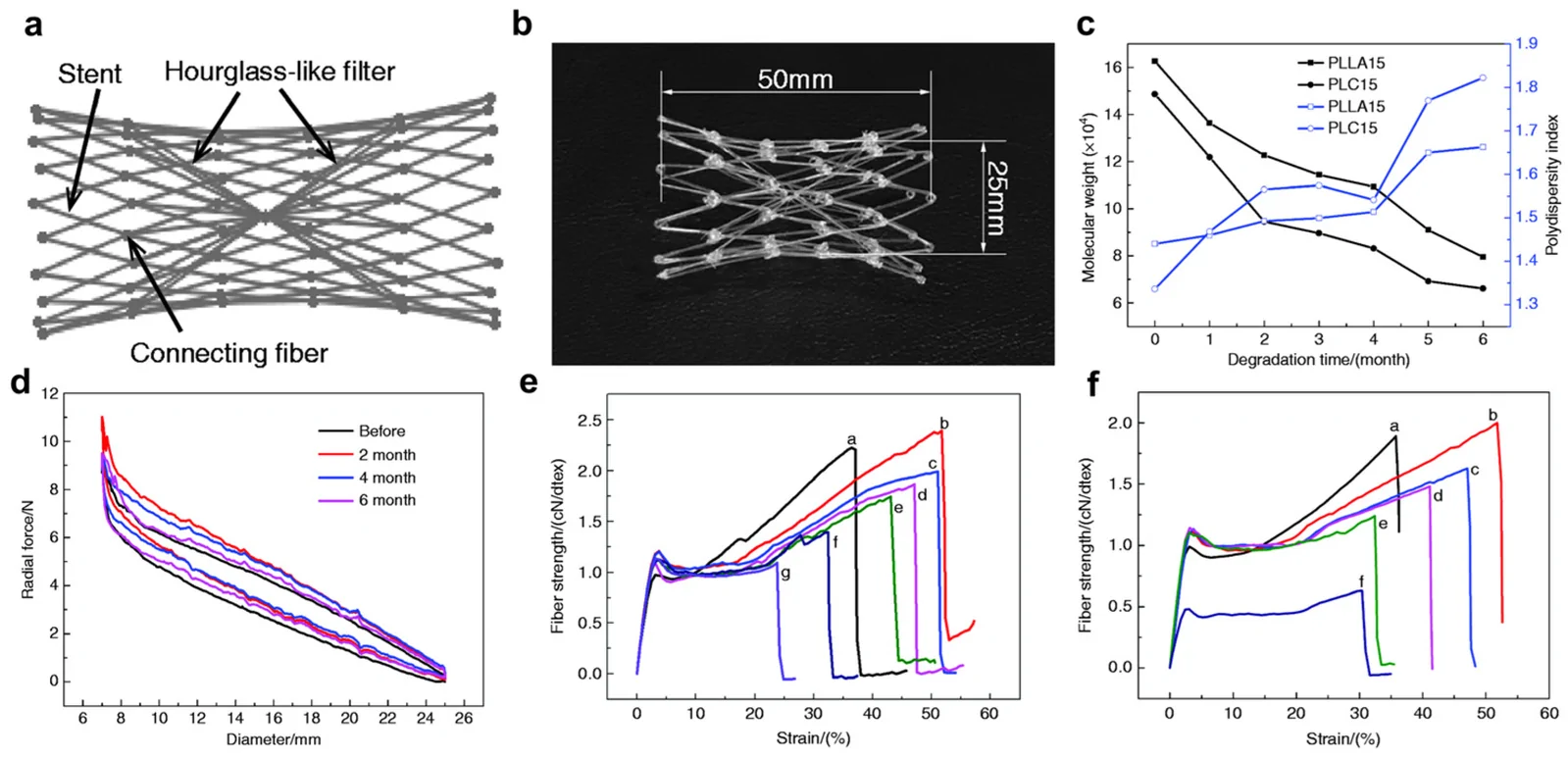
(**a**) Schematic illustration of the IVCF. (**b**) Photograph of the manually fabricated biodegradable IVCF. (**c**) Molecular weight (Mw) and polydispersity index (PDI) changes in PLLA15 and PLC15 fibers during degradation (solid symbols, Mw; open symbols, PDI). (**d**) Radial force of IVCFs after 0, 2, 4, and 6 months of in vitro degradation. (**e**) Tensile strength–strain curves of PLLA15 fibers at 0, 1, 2, 3, 4, 5, and 6 months of in vitro degradation, represented by curves a–g. (**f**) Tensile strength–strain curves of PLC15 fibers at 0, 1, 2, 3, 4, 5, and 6 months of in vitro degradation, represented by curves a–f. Reproduced from Ref. [77] with permission.

Yang et al. [77]. designed a polymeric filter in which the supporting framework and connecting fibers were fabricated from PLLA, while the hourglass-shaped filtering structure consisted of poly(L-lactide-co-e-caprolactone, 95/5 molar ratio) (PLC), as depicted in Figure 7a,b. By integrating polymers with different hydrolytic degradation behaviors into separate functional components, the filter was designed to undergo progressive structural resorption rather than simultaneous degradation of the entire device.

In vitro degradation studies confirmed that polymer composition effectively regulated the degradation sequence of individual filter components. As reflected by the degradation profiles in Figure 7c, PLC exhibited a substantially faster reduction in molecular weight than PLLA during degradation in phosphate-buffered saline at 37 °C, decreasing from approximately 1.5 × 10^5^ to 9.0 × 10^4^ within the first two months, whereas PLLA showed a more gradual decline over 6 months. This differential degradation was accompanied by distinct changes in mechanical properties. As shown in Figure 7d, the radial force of the IVCF initially increased during the first two months and subsequently decreased, remaining close to its original level at six months. Figure 7e,f further showed that PLC15 underwent a more pronounced decline in tensile strength and elongation than PLLA15, with the PLC15 fibers becoming unable to be stretched after approximately five months. Together, these results indicate that the faster degradation of PLC15 progressively reduced the mechanical integrity of the hourglass-like filter before the PLLA-based connecting and stent components, supporting a sequential loss of filter function while maintaining the structural support of the stent. Correspondingly, PLC fibers developed surface microcracks earlier and exhibited a greater reduction in glass transition temperature than PLLA, indicating accelerated molecular chain mobility, material softening and structural deterioration. These findings demonstrate that rational regulation of polymer composition enables preferential degradation of the filtering component while preserving the mechanical integrity of the supporting framework throughout the early implantation period.

The degradation sequence established by material selection was intended to maintain filtration performance while minimizing the structural risks associated with simultaneous polymer resorption. In an in vitro vena cava circulation model, the filter achieved thrombus capture efficiencies of 80% and 90% for simulated thrombi measuring 5 mm × 5 mm and 5 mm × 10 mm, respectively, demonstrating filtration performance comparable to that of conventional IVCFs. The sustained structural support provided by the PLLA framework was expected to maintain device stability during progressive degradation of the PLC filtering component, thereby reducing the risk of fragment release or transient vena cava obstruction that may accompany uncontrolled degradation of fully polymeric devices.

#### 4.2.2. Semi-Degradable Bioconvertible Filters

Semi-degradable bioconvertible filters are those in which the trapping structures are fabricated from rapidly degrading materials, whereas the main filter body is constructed from non-degradable nitinol. The permanent metallic framework provides durable mechanical support, whereas biodegradable polymeric components function as temporary degradation-sensitive triggers that regulate filter configuration. Rather than serving as load-bearing structures, the biodegradable polymers determine the duration of filtration, allowing thrombus protection to be terminated automatically after completion of the high-risk period while preserving the long-term mechanical stability of the metallic scaffold.

**Figure 8:**
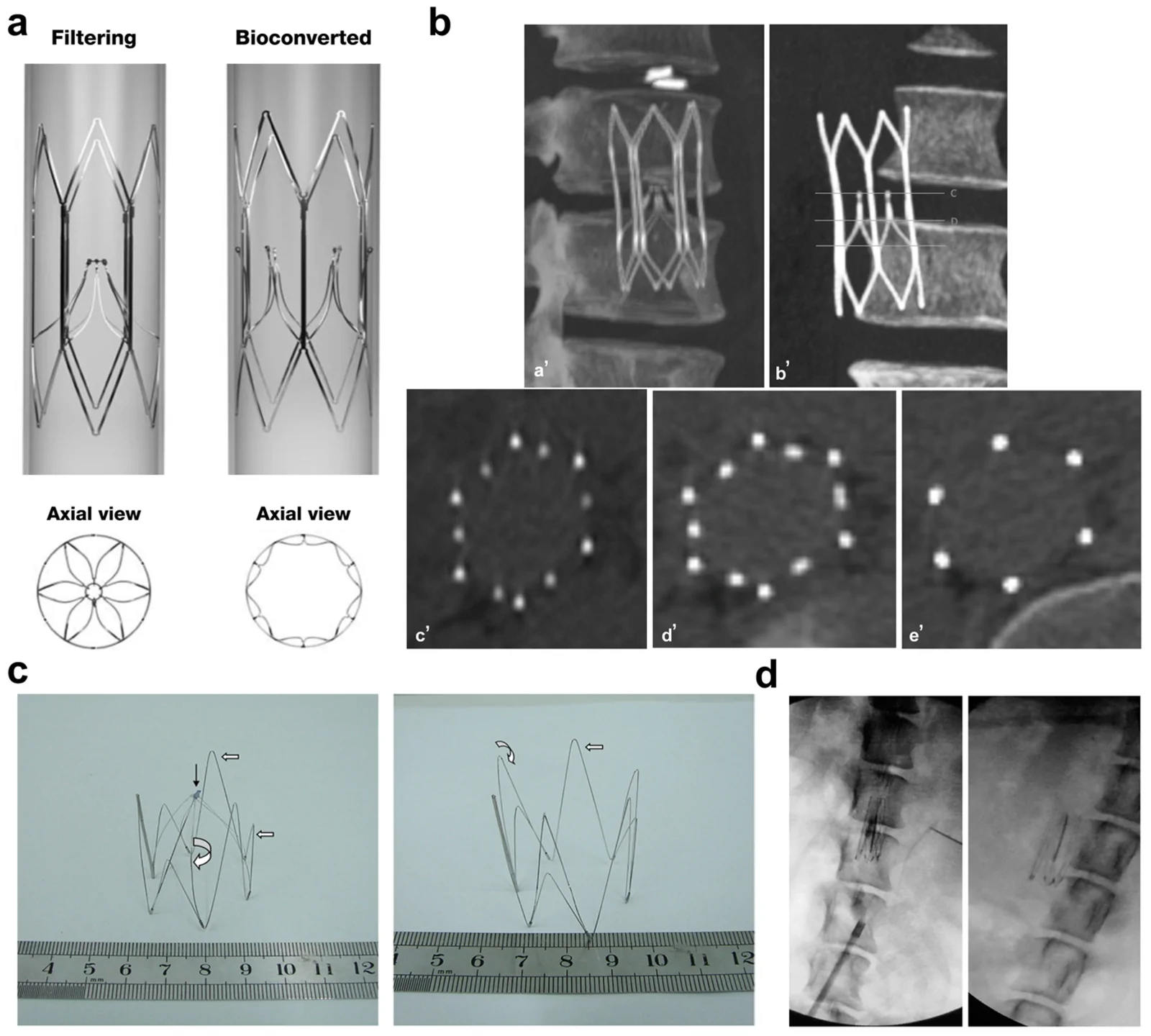
(**a**) Photographs of the Sentry IVCF in the filtering (**left**) and bioconverted (**right**) configurations, together with the corresponding axial-view schematics. In the filtering configuration, six pairs of filter arms are held at the center of the lumen by a bioabsorbable PPDO filament. Following hydrolytic degradation of the PPDO filament, the filter arms are released from the central cone and retract toward the IVC wall, where they become endothelialized, leaving the IVC lumen unobstructed. (**b**) Representative CT images of the Sentry IVCF obtained from a single patient. In panel (**b**), image a’ shows the device in the filtering configuration 1 month after implantation, whereas image b’ shows the bioconverted configuration at the 6-month follow-up. Images c’–e’ present the corresponding axial CT images acquired at the same follow-up time point. (**c**) Photographs of the self-converting filter (SCF) before (**left**) and after (**right**) conversion. Before conversion, the SCF consists of anchoring struts (white straight arrows), convertible struts (white curved arrows), and a biodegradable switch (black arrow). The anchoring struts comprise three long and three short inverted V-shaped struts, while the convertible struts are attached to the bases of the short anchoring struts to maintain stable positioning within the IVC. The filter has an overall length of 45 mm and a maximum diameter of 35 mm. (**d**) Representative cavographic images of the SCF. The left image shows appropriate self-positioning immediately after implantation, whereas the right image shows complete conversion 2 weeks after implantation. Panels (**a**,**b**) were reproduced from Ref. [78] with permission. Panels (**c**,**d**) were reproduced from Ref. [80] with permission.

The most clinically advanced example of this concept is the Sentry bioconvertible filter [78,79]. As shown in Figure 8a, the device consists of a laser-cut nitinol cylindrical framework with six pairs of filtering arms restrained at the center of the IVC by a bioabsorbable PPDO filament. Following implantation, hydrolytic degradation of the filament releases the filtering arms, which retract toward the caval wall, converting the device from a filtering to a non-filtering configuration without requiring secondary retrieval. Preclinical studies demonstrated stable incorporation of the frame and retracted filter arms into the caval wall while maintaining caval patency.

In the prospective multicenter SENTRY trial involving 129 patients with documented DVT or PE, or at temporary risk of developing DVT or PE with contraindications to anticoagulation, the device provided effective protection against PE throughout the intended 60-day protection period, with no new symptomatic PE during this interval. Representative CT images in Figure 8b demonstrate the transition from the filtering configuration at 1 month to the bioconverted configuration at the 6-month follow-up. Clinical success at 6 months was achieved in 97.4% of evaluable patients, while successful bioconversion was confirmed in 95.7% (110/115) at 6 months and 96.4% (106/110) at 12 months. Throughout 12 months of follow-up, no filter tilting, migration, embolization, fracture, caval perforation, or filter-related death was reported. The only reported symptomatic filter complication involving the filter was symptomatic caval thrombosis in two patients during the first month, both successfully treated without recurrence.

Two-year follow-up confirmed sustained caval patency, with successful bioconversion in 96.5% of evaluable patients and no evidence of late-stage IVC obstruction, thrombosis after bioconversion, or thrombogenicity associated with the retracted filter arms. Two new symptomatic nonfatal pulmonary emboli occurred between 12 and 24 months, but both events occurred after confirmed bioconversion and were adjudicated as unrelated to the device or procedure. These findings suggest that successful device bioconversion restored the non-filtering configuration while maintaining a low incidence of device-related complications, providing an alternative to retrievable filters that frequently remain indwelling and are associated with time-dependent complications.

Gao et al. [80]. proposed an early self-convertible IVCF incorporating a biodegradable poly(caprolactone-co-L-lactide) (PCLA75) switch to eliminate the need for secondary retrieval procedures. As presented in Figure 8c, the device consisted of anchoring struts, convertible struts, and a biodegradable switch, in which the PCLA75 component bound the apices of the convertible struts to form the filtering configuration. As the switch progressively degraded, the convertible struts returned to their original position against the caval wall, thereby terminating filtration while leaving the supporting framework in situ. Accelerated in vitro degradation using a circulating lipase solution (20 IU mL^−1^, pH 8.0) resulted in complete conversion of all filters within 27.7 h ± 3.8 without filter tilting or fracture. The in vivo feasibility was further evaluated in five adult beagle dogs weighing 8–12 kg, with representative cavographic images in Figure 8d confirming appropriate self-positioning immediately after implantation and complete bioconversion after 2 weeks. All filters were successfully implanted into the infrarenal IVC and achieved complete bioconversion within 2 weeks without fracture.

In vitro thrombus-capture experiments further demonstrated a mean capture efficiency of 75.5%, reaching 92% under the optimized condition of a 22 mm caval diameter, 5 mm clot diameter, and upright orientation. Capture efficiency decreased with increasing caval diameter, smaller clot size, and repeated clot accumulation. Recognizing that spontaneous degradation could trigger conversion irrespective of residual thrombus burden, the authors proposed future incorporation of an externally controlled protective coating to delay degradation until filtration was no longer required. The study identified uncontrolled post-implantation degradation as a major limitation of self-convertible filters and proposed externally triggered initiation of switch degradation to synchronize bioconversion with the completion of thromboembolic protection, providing an important conceptual basis for subsequent convertible filter designs.

Compared with fully degradable bioconvertible filters, semi-degradable bioconvertible filters leave a non-degradable main body in the lumen after the tip-trapping structures degrade and convert into a stent-like configuration. However, as a widely used endovascular device in clinical practice, the stent exhibits far fewer long-term complications after endothelialization than conventional non-degradable filters. Owing to the use of nitinol, a material already extensively employed in clinical settings for the main body, semi-degradable bioconvertible filters have advanced into clinical trials ahead of their fully degradable counterparts. Recently, we have been developing a novel convertible filter that incorporates a retrievable device alongside the conversion feature. In this design, after the short-term therapeutic task is completed, the filter can first be attempted for retrieval; should retrieval fail, it automatically converts into a stent-like structure in vivo. Evidently, this strategy combines the advantages of both retrievable and convertible filters, thereby achieving a better balance between short-term therapeutic efficacy and long-term safety.

### 4.3. Multilayer Structural Design

Multilayer structural design has been explored in biodegradable stents to modulate the temporal behavior of different polymer layers [81], and this design strategy can likewise be adopted in filter design. It provides one route to this objective by separating mechanical retention from biological regulation. In biodegradable vascular implants, a mechanically competent metallic framework can preserve load bearing capacity during the high risk phase, whereas a degradable bioactive polymer coating modulates early corrosion, interfacial bioactivity, and subsequent endothelialization.

Material-gradient design further improves the precision of degradation regulation by coupling degradation kinetics with local mechanical requirements. Through spatial modulation of alloy composition, crystallinity, or microstructure, degradation can be preferentially initiated in mechanically noncritical regions while preserving the integrity of primary load-bearing components, thereby reducing abrupt mechanical deterioration and the risk of fragment-induced embolization. In the broader context of biodegradable metallic vascular stents, Yang et al. [82]. reported electrodeposited Fe–Zn alloy microtube with a compositionally regulated ultrafine-grained microstructure with grain sizes of several hundred nanometers, which suppressed localized galvanic corrosion and promoted more homogeneous degradation while maintaining an ultimate tensile strength of approximately 430 MPa, an elongation of about 16%, and endothelial cell viability above 90%. These findings indicate that material composition and microstructural characteristics can influence degradation uniformity, mechanical retention, and cytocompatibility. Although demonstrated in a biodegradable vascular stent rather than an IVCF, these findings may offer useful considerations for material selection and design in future biodegradable IVCFs.

### 4.4. Radiopacity Enhancement Strategies

Radiographic visualization is essential for accurate deployment, postoperative surveillance and degradation assessment of biodegradable IVCFs. This requirement applies to both polymeric and biodegradable metallic filters, although the underlying causes differ. Polymeric materials such as PPDO possess intrinsically low atomic numbers and therefore exhibit negligible X-ray attenuation [83], whereas biodegradable metals, particularly magnesium-based alloys, gradually lose radiographic contrast during corrosion because of their low density and continuous material resorption. Consequently, radiopacity enhancement has become an important design consideration for biodegradable IVCFs, enabling precise localization throughout the functional lifetime of the device while facilitating evaluation of degradation behavior.

Current strategies primarily rely on incorporating high atomic number nanoparticles into the filter matrix to increase X-ray attenuation through enhanced photoelectric absorption. Gold nanoparticles were among the earliest radiopaque additives investigated. Gold-loaded PPDO filters exhibited approximately one order of magnitude higher micro-CT signal than the unmodified polymer, while maintaining stable imaging during the degradation period. However, the high material cost has limited their translational potential despite excellent radiopacity and biocompatibility [84,85].

Subsequent studies have focused on alternative contrast agents with improved cost effectiveness or multimodal imaging capability. Gadolinium nanoparticles substantially enhanced the radiopacity of PPDO-based IVCFs, achieving an in vitro CT attenuation of 2823 ± 51 HU, while maintaining clot-trapping efficacy and sustained radiopacity during 12 weeks of in vivo monitoring [86]. Tungsten nanoparticles have emerged as a practical alternative because they combine high radiopacity with relatively low cost. PPDO composites containing optimized tungsten loading achieved CT values of approximately 200 HU, maintained stable radiographic visibility throughout a 12-week porcine study, and preserved more than 90% of their original tensile strength after ethylene oxide sterilization [33].

Collectively, current evidence indicates that high atomic number nanoparticles substantially improve the visibility of biodegradable IVCFs without markedly compromising degradation behavior or mechanical integrity. Future development should extend beyond maximizing radiographic contrast toward integrating long-term imaging stability, degradation-dependent signal evolution, mechanical reliability and biological safety, thereby enabling continuous image-guided evaluation throughout the entire service life of biodegradable filters.

## 5. Conclusions and Prospects

The principal advantage of biodegradable IVCFs lies in their ability to provide temporary thromboembolic protection without leaving a permanent intravascular implant, thereby addressing some of the long-term complications associated with conventional metallic filters [87,88,89]. Their degradable nature also creates opportunities to allow transient mechanical protection while potentially reducing the need for permanent implantation and subsequent retrieval. However, these advantages are inseparable from several material- and device-specific limitations [90]. Compared with permanent metallic filters, biodegradable systems generally face tighter constraints in simultaneously achieving sufficient radial support, fatigue resistance, deployment performance and predictable degradation under dynamic venous loading [91,92]. Their performance is further constrained by the coupling among degradation kinetics, mechanical retention, and biological response, such that premature structural loss may compromise thrombus capture, whereas delayed resorption, nonuniform fragmentation, or accumulation of degradation byproducts may offset the intended safety benefit. Therefore, the core challenge in biodegradable IVCF development is not merely to replace permanent materials with degradable ones but to establish a functional matching among filtration efficacy, structural persistence, degradation pathway, and long-term biosafety during the period when effective thrombus interception is clinically required. Future material selection should consider not only biodegradability and mechanical strength but also degradation kinetics, fatigue resistance, processability, and functional lifetime. Materials with complementary mechanical and degradation characteristics may be combined to better align device persistence with the thromboembolic risk window, providing a rational basis for bioconvertible and fully biodegradable filter systems.

The microenvironmental effects of degradation byproducts and the long-term safety of residual fragments remain critical constraints on the clinical translation of biodegradable materials [93]. During the microscopic degradation of aliphatic polyesters, the release of acidic oligomers has been reported to alter local pH and induce inflammatory responses in biodegradable vascular implants, which may adversely affect endothelial function [94,95,96]. Particularly during the later stages of degradation, the generation and detachment of residual filter fragments may raise safety concerns, especially if their size and persistence are not adequately controlled, as such fragments could potentially enter the pulmonary circulation and their biological consequences remain incompletely understood [97]. Future evaluation should therefore integrate molecular degradation, mechanical retention, fragment formation, degradation products, and local tissue responses to provide a more comprehensive assessment of functional degradation [98].

From a structural perspective, existing biodegradable IVCF designs suggest that the filtering and anchoring components do not necessarily require identical mechanical or degradation characteristics. Component-specific or multicomponent designs may therefore provide opportunities to retain adequate thrombus interception and vessel fixation while limiting unnecessary long-term material persistence [77]. However, the effects of such structural modifications on filtration efficiency, venous flow, mechanical stability, and tissue interaction require direct experimental validation rather than assumption [99]. Future studies should consequently focus on establishing consistent relationships among degradation, mechanical performance, fragment fate, thrombus capture, and vascular responses under physiologically relevant conditions.

The evidence discussed in this review should be interpreted according to its source. Direct biodegradable IVCF studies provide the primary evidence for device-specific performance, including mechanical retention, thrombus capture, degradation behavior, biocompatibility, and clinical outcomes. In contrast, evidence from bioresorbable vascular stents and other vascular implants mainly informs material selection, degradation mechanisms, and structural design strategies that have not yet been systematically evaluated in IVCFs. Accordingly, findings from related vascular implants are considered supportive evidence rather than direct validation of IVCF performance. This distinction is particularly important for biodegradable metallic materials, multilayer architectures, and material-gradient design, for which IVCF-specific evidence remains limited.

In summary, biodegradable IVCFs represent a shift from permanent implantation toward transient vascular protection. Future progress will depend primarily on rational material selection and function-oriented structural design, with the degradation profile and mechanical persistence of each component matched to its intended role and the clinically required protection period. More consistent assessment of mechanical integrity, degradation, fragment formation, hemodynamics, and tissue response would improve comparison across studies and help define performance requirements for future device development [100,101].

## Figures and Tables

**Table 1:** Representative biodegradable polymeric IVCFs.

Parameter	PPDO	PLA	PGA+PCL
Degradation	Functional ≥5–6 weeks; intact ~12 weeks; near-complete resorption ~32 weeks	In-vitro degradation studied to 3 months; in-vivo lifetime unknown	PGA~6 weeks; PCL > 24 months
Mechanical performance	79% strength retained at 5 weeks; adequate early support	Good radial support and self-expansion in vitro	Low radial force; inadequate anchoring
Thrombus filtration	Effective in vivo; no PE in animal studies	~90% capture in vitro	Not formally evaluated in vivo
Animal studies	Multiple swine studies; up to randomized GLP comparison	None	Beagles, *n* = 10
Clinical studies	First-in-human, 8 implanted patients	None	None
Major strength	Best balance of support–degradation timing; strongest translational evidence	Favorable bench performance	Rapid disappearance of filtering component
Major limitation	Larger clinical trials still required	No in-vivo validation	100% migration; slow PCL persistence and tissue reaction
Reference(s)	[59,60,61,62]	[63]	[64]

## Data Availability

The original contributions presented in this study are included in the article. Further inquiries can be directed to the corresponding authors.

## Abbreviations

The following abbreviations are used in this manuscript:

VTE	Venous thromboembolism
PE	Pulmonary embolism
IVCF	Inferior vena cava filter
DVT	Deep vein thrombosis
IVC	Inferior vena cava
Nitinol	Nickel-titanium alloys
PCL	Poly(ε-caprolactone)
PGA	Poly(glycolic acid)
PLA	Poly(lactic acid)
PLLA	Poly(L-lactic acid)
PDLLA	Poly(D,L-lactic acid)
PPDO	Poly(p-dioxanone)
CT	Computed tomography
H&E	Hematoxylin–eosin
SEM	Scanning electron microscopy
4D	Four-dimensional
PGSA-co-HEMA	Poly(glycerol sebacate) acrylate-co-hydroxyethyl methacrylate
PHEMA	Poly(2-Hydroxyethyl methacrylate)
PGSA	Poly(glycerol sebacate) acrylate
DSC	Differential scanning calorimetry
PLC	Poly(L-lactide-co-e-caprolactone)
Mw	Molecular weight
PDI	Polydispersity index
PCLA	Poly(caprolactone-co-L-lactide)
SCF	Self-converting filter

## References

[1] Thaler J., Pabinger I., Ay C. (2015). Anticoagulant Treatment of Deep Vein Thrombosis and Pulmonary Embolism: The Present State of the Art. Front. Cardiovasc. Med..

[2] Beckman M.G., Hooper W.C., Critchley S.E., Ortel T.L. (2010). Venous Thromboembolism: A Public Health Concern. Am. J. Prev. Med..

[3] Pastori D., Cormaci V.M., Marucci S., Franchino G., Del Sole F., Capozza A., Fallarino A., Corso C., Valeriani E., Menichelli D. (2023). A Comprehensive Review of Risk Factors for Venous Thromboembolism: From Epidemiology to Pathophysiology. Int. J. Mol. Sci..

[4] Konstantinides S.V., Meyer G., Becattini C., Bueno H., Geersing G.J., Harjola V.P., Huisman M.V., Humbert M., Jennings C.S., Jiménez D. (2020). 2019 ESC Guidelines for the Diagnosis and Management of Acute Pulmonary Embolism Developed in Collaboration with the European Respiratory Society (ERS). Eur. Heart J..

[5] Wang X., Wang H., Qian H., Su H., Lou D., Tian L., Chen D., Ding H., Fan F. (2025). Design and Performance Analysis of a New Inferior Vena Cava Filter. Comput. Methods Biomech. Biomed. Eng..

[6] Kim B.-J., Lee C. (2024). Optimizing Inferior Vena Cava Filter Design: A Computational Fluid Dynamics Study on Strut Configuration for Enhanced Hemodynamic Performance and Thrombosis Reduction. Heliyon.

[7] Prince M.R., Salzman E.W., Schoen F.J., Palestrant A.M., Simon M. (1988). Local Intravascular Effects of the Nitinol Wire Blood Clot Filter. Investig. Radiol..

[8] Kerwin A.J., Lombardo S., Haut E.R., Knudson M.M. (2025). For The Clott Study Group. Variability of Prophylactic Inferior Vena Cava Filter Use in Young Trauma Patients: Analysis of the Consortium of Leaders in the Study of Traumatic Thromboembolism Database. J. Am. Coll. Surg..

[9] He J., Wang Z., Zhou Y.X., Ni H., Sun X., Xue J., Chen S., Wang S., Niu M. (2022). The Application of Inferior Vena Cava Filters in Orthopaedics and Current Research Advances. Front. Bioeng. Biotechnol..

[10] Ray C.E., Kaufman J.A. (1996). Complications of inferior vena cava filters. Abdom. Imaging..

[11] Harvey J.J., Hopkins J., McCafferty I.J., Jones R.G. (2013). Inferior Vena Cava Filters: What Radiologists Need to Know. Clin. Radiol..

[12] Deso S.E., Idakoji I.A., Kuo W.T. (2016). Evidence-Based Evaluation of Inferior Vena Cava Filter Complications Based on Filter Type. Semin. Interv. Radiol..

[13] Covello B., Radvany M. (2022). Back to the Basics: Inferior Vena Cava Filters. Semin. Interv. Radiol..

[14] DeYoung E., Minocha J. (2016). Inferior Vena Cava Filters: Guidelines, Best Practice, and Expanding Indications. Semin. Interv. Radiol..

[15] Shayan M., Chun Y. (2015). An overview of thin film nitinol endovascular devices. Acta Biomater..

[16] Sarosiek S., Crowther M., Sloan J.M. (2013). Indications, Complications, and Management of Inferior Vena Cava Filters: The Experience in 952 Patients at an Academic Hospital with a Level I Trauma Center. JAMA Intern. Med..

[17] Grewal S., Chamarthy M.R., Kalva S.P. (2016). Complications of Inferior Vena Cava Filters. Cardiovasc. Diagn. Ther..

[18] Kearon C., Akl E.A., Ornelas J., Blaivas A., Jimenez D., Bounameaux H., Huisman M., King C.S., Morris T.A., Sood N. (2016). Antithrombotic Therapy for VTE Disease: CHEST Guideline and Expert Panel Report. Chest.

[19] Marron R.M., Rali P., Hountras P., Bull T.M. (2020). Inferior Vena Cava Filters: Past, Present, and Future. Chest.

[20] Morrow K.L., Bena J., Lyden S.P., Parodi E., Smolock C.J. (2020). Factors Predicting Failure of Retrieval of Inferior Vena Cava Filters. J. Vasc. Surg. Venous Lymphat. Disord..

[21] Rodriguez A.K., Goel A., Gorantla V.R. (2024). Complications Associated with Inferior Vena Cava Filter Retrieval: A Systematic Review. Cureus.

[22] Ferro E.G., Mackel J.B., Kramer R.D., Torguson R., Whatley E.M., O’Connell G., Pullin B., Watson N.W., Li S., Song Y. (2024). Postmarketing Surveillance of Inferior Vena Cava Filters among US Medicare Beneficiaries: The SAFE-IVC Study. JAMA.

[23] Angel L.F., Tapson V., Galgon R.E., Restrepo M.I., Kaufman J. (2011). Systematic Review of the Use of Retrievable Inferior Vena Cava Filters. J. Vasc. Interv. Radiol..

[24] Garcia-Godoy F., Collins T., Sacks D., Vasas S., Sarani B. (2011). Retrieval of Inferior Vena Cava Filters After Prolonged Indwelling Time. Arch. Intern. Med..

[25] Williams A.O., Sridharan N., Rojanasarot S., Chaer R., Anderson N., Wifler W., Jaff M.R. (2022). Population-Based Disparities in Inferior Vena Cava Filter Procedures among Medicare Enrollees with Acute Venous Thromboembolism. J. Am. Coll. Radiol..

[26] Wong J.K.L., Tan M., Bakhshayesh P. (2021). A Review of Preclinical Absorbable Inferior Vena Cava Filters. J. Vasc. Surg. Venous Lymphat. Disord..

[27] Benedetti R., Marino S., Tangianu F., Imberti D. (2024). Inferior Vena Cava Filters: A Clinical Review and Future Perspectives. J. Clin. Med..

[28] Dria S.J., Eggers M.D. (2016). In Vitro Evaluation of Clot Capture Efficiency of an Absorbable Vena Cava Filter. J. Vasc. Surg. Venous Lymphat. Disord..

[29] Kaufman J.A., Barnes G.D., Chaer R.A., Cuschieri J., Eberhardt R.T., Johnson M.S., Kuo W.T., Murin S., Patel S., Rajasekhar A. (2020). Society of Interventional Radiology Clinical Practice Guideline for Inferior Vena Cava Filters in the Treatment of Patients with Venous Thromboembolic Disease: Developed in collaboration with the American College of Cardiology, American College of Chest Physicians, American College of Surgeons Committee on Trauma, American Heart Association, Society for Vascular Surgery, and Society for Vascular Medicine. J. Vasc. Interv. Radiol..

[30] Niu L., Liu Z., Geng X., Zhong X., Zhao H., Zhang H., Xi J., Feng Z., Zhang F., Ye L. (2022). Anti-Coagulation and Anti-Hyperplasia Coating for Retrievable Vena Cava Filters by Electrospraying and Their Performance in Vivo. Int. J. Pharm..

[31] Zhang H., Zhong X., Wen J., Xi J., Feng Z., Liu Z., Ye L. (2024). Hydrogel Coating Containing Heparin and Cyclodextrin/Paclitaxel Inclusion Complex for Retrievable Vena Cava Filter towards High Biocompatibility and Easy Removal. Int. J. Biol. Macromol..

[32] Melancon S.T., San Valentin E.M., Bolinas D.K.M., Bernardino M.R., Mishra A., Canlas G., Chintalapani G., Jacobsen M.C., Barcena A.J.R., Huang S.Y. (2025). Fabrication of Radiopaque, Drug-Loaded Resorbable Polymer for Medical Device Development. Polymers.

[33] San Valentin E.M., Damasco J.A., Bernardino M., Court K.A., Godin B., Canlas G.M., Melancon A., Chintalapani G., Jacobsen M.C., Norton W. (2024). Image-Guided Deployment and Monitoring of a Novel Tungsten Nanoparticle-Infused Radiopaque Absorbable Inferior Vena Cava Filter in a Swine Model. J. Vasc. Interv. Radiol..

[34] Vert M. (2005). Aliphatic Polyesters: Great Degradable Polymers That Cannot Do Everything. Biomacromolecules.

[35] Nair L.S., Laurencin C.T. (2007). Biodegradable Polymers as Biomaterials. Prog. Polym. Sci..

[36] Ulery B.D., Nair L.S., Laurencin C.T. (2011). Biomedical Applications of Biodegradable Polymers. J. Polym. Sci. Part B Polym. Phys..

[37] Manavitehrani I., Fathi A., Badr H., Daly S., Negahi Shirazi A., Dehghani F. (2016). Biomedical Applications of Biodegradable Polyesters. Polymers.

[38] Ang H.Y., Huang Y.Y., Lim S.T., Wong P., Joner M., Foin N. (2017). Mechanical Behavior of Polymer-Based vs. Metallic-Based Bioresorbable Stents. J. Thorac. Dis..

[39] Qiu T., Zhao L. (2018). Research into Biodegradable Polymeric Stents: A Review of Experimental and Modelling Work. Vessel Plus.

[40] Li Y., Chu Z., Li X., Ding X., Guo M., Zhao H., Yao J., Wang L., Cai Q., Fan Y. (2017). The Effect of Mechanical Loads on the Degradation of Aliphatic Biodegradable Polyesters. Regen. Biomater..

[41] Woodard L.N., Grunlan M.A. (2018). Hydrolytic Degradation and Erosion of Polyester Biomaterials. ACS Macro Lett..

[42] Woodruff M.A., Hutmacher D.W. (2010). The Return of a Forgotten Polymer—Polycaprolactone in the 21st Century. Prog. Polym. Sci..

[43] Samantaray P.K., Little A., Haddleton D.M., McNally T., Tan B., Sun Z., Huang W., Ji Y., Wan C. (2020). Poly(Glycolic Acid) (PGA): A Versatile Building Block Expanding High Performance and Sustainable Bioplastic Applications. Green Chem..

[44] Hussain M., Khan S.M., Shafiq M., Abbas N. (2024). A Review on PLA-Based Biodegradable Materials for Biomedical Applications. Giant.

[45] Li S., McCarthy S. (1999). Influence of Crystallinity and Stereochemistry on the Enzymatic Degradation of Poly(Lactide)s. Macromolecules.

[46] Gorrasi G., Pantani R., Di Lorenzo M.L., Androsch R. (2017). Hydrolysis and Biodegradation of Poly(Lactic Acid). Synthesis, Structure and Properties of Poly(Lactic Acid).

[47] Weir N.A., Buchanan F.J., Orr J.F., Farrar D.F., Boyd A. (2004). Processing, Annealing and Sterilisation of Poly-L-Lactide. Biomaterials.

[48] Farah S., Anderson D.G., Langer R. (2016). Physical and Mechanical Properties of PLA, and Their Functions in Widespread Applications: A Comprehensive Review. Adv. Drug Deliv. Rev..

[49] Vollrath A., Kretzer C., Beringer-Siemers B., Shkodra B., Czaplewska J.A., Bandelli D., Stumpf S., Hoeppener S., Weber C., Werz O. (2021). Effect of Crystallinity on the Properties of Polycaprolactone Nanoparticles Containing the Dual FLAP/mPEGS-1 Inhibitor BRP-187. Polymers.

[50] Dias J.R., Sousa A., Augusto A., Bártolo P.J., Granja P.L. (2022). Electrospun Polycaprolactone (PCL) Degradation: An in Vitro and in Vivo Study. Polymers.

[51] Erdal N.B., Lando G.A., Yadav A., Srivastava R.K., Hakkarainen M. (2020). Hydrolytic Degradation of Porous Crosslinked Poly(ε-Caprolactone) Synthesized by High Internal Phase Emulsion Templating. Polymers.

[52] Kim G., Gavande V., Shaikh V., Lee W.-K. (2023). Degradation Behavior of Poly(Lactide-Co-Glycolide) Monolayers Investigated by Langmuir Technique: Accelerating Effect. Molecules.

[53] Roberts C.T., Grunlan M.A. (2025). Biodegradable Polyesters: Approaches to Increase Degradation Rates for Biomedical Applications. ACS Macro Lett..

[54] Pawlak A. (2019). The Entanglements of Macromolecules and Their Influence on the Properties of Polymers. Macromol. Chem. Phys..

[55] Gleadall A., Pan J., Kruft M.-A., Kellomäki M. (2014). Degradation Mechanisms of Bioresorbable Polyesters. Part 1. Effects of Random Scission, End Scission and Autocatalysis. Acta Biomater..

[56] Pawlak A., Krajenta J. (2024). Entanglements of Macromolecules and Their Influence on Rheological and Mechanical Properties of Polymers. Molecules.

[57] Eckel F., Van Opdenbosch D., Schandl K.S., Zollfrank C. (2024). Dependency of Tensile Properties and Biodegradation on Molecular Mass during Hydrolysis of Poly(Butylene Succinate). npj Mater. Degrad..

[58] Eggers M.D., Reitman C.A. (2012). In Vitro Analysis of Polymer Candidates for the Development of Absorbable Vascular Filters. J. Vasc. Interv. Radiol..

[59] Eggers M.D., McArthur M.J., Figueira T.A., Abdelsalam M.E., Dixon K.P., Pageon L.R., Wallace M.J., Huang S.Y. (2015). Pilot in Vivo Study of an Absorbable Polydioxanone Vena Cava Filter. J. Vasc. Surg. Venous Lymphat. Disord..

[60] Huang S.Y., Eggers M., McArthur M.J., Dixon K.A., McWatters A., Dria S., Hill L.R., Melancon M.P., Steele J.R., Wallace M.J. (2017). Safety and Efficacy of an Absorbable Filter in the Inferior Vena Cava to Prevent Pulmonary Embolism in Swine. Radiology.

[61] Eggers M., Rousselle S.D., Urtz M., Albright R., Will A., Jourden B., Godshalk C., Dria S., Huang S., Steele J. (2019). Randomized Controlled Study of an Absorbable Vena Cava Filter in a Porcine Model. J. Vasc. Interv. Radiol..

[62] Elizondo G., Eggers M., Falcon M., Trevino M., Marrufo R., Perez C., Nunez E., Moreno R., Mitchell A., Sheppard A. (2020). First-in-Human Study with Eight Patients Using an Absorbable Vena Cava Filter for the Prevention of Pulmonary Embolism. J. Vasc. Interv. Radiol..

[63] Cui H., Chen T., Song C. (2018). Preparation and Properties of a Biodegradable Inferior Vena Cava Filter. Polym. Adv. Technol..

[64] Zhang F., Li H., Liang G., Zhang H. (2017). Development and Evaluation of a New Biodegradable Vena Cava Filter in a Canine Model. Asian J. Surg..

[65] Morales J.P., Li X., Irony T.Z., Ibrahim N.G., Moynahan M., Cavanaugh K.J. (2013). Decision analysis of retrievable inferior vena cava filters in patients without pulmonary embolism. J. Vasc. Surg. Venous Lymphat. Disord..

[66] Huang W.M., Ding Z., Wang C.C., Wei J., Zhao Y., Purnawali H. (2010). Shape Memory Materials. Mater. Today.

[67] Lendlein A., Langer R. (2002). Biodegradable, Elastic Shape-Memory Polymers for Potential Biomedical Applications. Science.

[68] Yakacki C.M., Shandas R., Lanning C., Rech B., Eckstein A., Gall K. (2007). Unconstrained Recovery Characterization of Shape-Memory Polymer Networks for Cardiovascular Applications. Biomaterials.

[69] Serrano M.C., Ameer G.A. (2012). Recent Insights into the Biomedical Applications of Shape-Memory Polymers. Macromol. Biosci..

[70] Zhao W., Liu L., Zhang F., Leng J., Liu Y. (2019). Shape Memory Polymers and Their Composites in Biomedical Applications. Mater. Sci. Eng. C.

[71] Peterson G.I., Dobrynin A.V., Becker M.L. (2017). Biodegradable Shape Memory Polymers in Medicine. Adv. Healthc. Mater..

[72] Qu R., Zhou D., Guo T., He W., Cui C., Zhou Y., Zhang Y., Tang Z., Zhang X., Wang Q. (2023). 4D Printing of Shape Memory Inferior Vena Cava Filters Based on Copolymer of Poly(Glycerol Sebacate) Acrylate-Co-Hydroxyethyl Methacrylate (PGSA-HEMA). Mater. Des..

[73] Choudhury S., Joshi A., Baghel V.S., Ananthasuresh G.K., Asthana S., Homer-Vanniasinkam S., Chatterjee K. (2024). Design-encoded dual shape-morphing and shape-memory in 4D printed polymer parts toward cellularized vascular grafts. J. Mater. Chem. B.

[74] Choudhury S., Patil P.M., Rehman T., Pemmaraju D.B., Chatterjee K. (2026). NIR-responsive 4D-printed shape memory polymer composites for imaging-guided deployable bio-medical constructs. Mater. Today Chem..

[75] Coleman J.J., Zarzaur B.L., Katona C.W., Plummer Z.J., Johnson L.S., Fecher A., O’Rear J.M., Feliciano D.V., Rozycki G.S. (2015). Factors Associated with Pulmonary Embolism within 72 Hours of Admission after Trauma: A Multicenter Study. J. Am. Coll. Surg..

[76] Kim Y.-J., Choi D.-H., Ahn S., Sohn C.H., Seo D.-W., Kim W.Y. (2016). Timing of Pulmonary Embolisms in Femur Fracture Patients: Incidence and Outcomes. J. Trauma Acute Care Surg..

[77] Yang C., Ma F., Gao C., Kang Y., Zhang G., Liu P., Jiang H., Chang Z. (2019). Design and Evaluation of a Novel Biodegradable Inferior Vena Cava Filter. J. Biomater. Appl..

[78] Dake M.D., Murphy T.P., Krämer A.H., Darcy M.D., Sewall L.E., Curi M.A., Johnson M.S., Arena F., Swischuk J.L., Ansel G.M. (2018). One-Year Analysis of the Prospective Multicenter SENTRY Clinical Trial: Safety and Effectiveness of the Novate Sentry Bioconvertible Inferior Vena Cava Filter. J. Vasc. Interv. Radiol..

[79] Dake M.D., Murphy T.P., Krämer A.H., Darcy M.D., Sewall L.E., Curi M.A., Johnson M.S., Arena F., Swischuk J.L., Ansel G.M. (2020). Final Two-Year Outcomes for the Sentry Bioconvertible Inferior Vena Cava Filter in Patients Requiring Temporary Protection from Pulmonary Embolism. J. Vasc. Interv. Radiol..

[80] Gao X., Zhang J., Chen B., Yu H., Li J., Zhang S., Feng Z., Ye L., Han J. (2011). A New Self-Convertible Inferior Vena Cava Filter: Experimental in-Vitro and in-Vivo Evaluation. J. Vasc. Interv. Radiol..

[81] Wang X., Venkatraman S.S., Boey F.Y.C., Loo J.S.C., Tan L.P. (2006). Controlled Release of Sirolimus from a Multilayered PLGA Stent Matrix. Biomaterials.

[82] Yang S., Yu F., Wang W., Gao Y., Zhang Z., Wang J., Miao P., Hao S. (2026). Biodegradable Fe-Zn Alloy Microtubes with Good Mechanical Properties, Degradability, and Cytocompatibility Prepared by Electrodeposition. Adv. Funct. Mater..

[83] Singhana B., Chen A., Slattery P., Yazdi I.K., Qiao Y., Tasciotti E., Wallace M., Huang S., Eggers M., Melancon M.P. (2015). Infusion of Iodine-Based Contrast Agents into Poly(p-Dioxanone) as a Radiopaque Resorbable IVC Filter. J. Mater. Sci. Mater. Med..

[84] Tian L., Lee P., Singhana B., Chen A., Qiao Y., Lu L., Martinez J.O., Tasciotti E., Melancon A., Huang S. (2017). Radiopaque Resorbable Inferior Vena Cava Filter Infused with Gold Nanoparticles. Sci. Rep..

[85] Huang S.Y., Damasco J.A., Tian L., Lu L., Perez J.V.D., Dixon K.A., Williams M.L., Jacobsen M.C., Dria S.J., Eggers M.D. (2020). In Vivo Performance of Gold Nanoparticle-Loaded Absorbable Inferior Vena Cava Filters in a Swine Model. Biomater. Sci..

[86] San Valentin E.M., Damasco J., Bernardino M., Mishra A., Barcena A.J.R., Bolinas D.K., Canlas G.M., Jacobsen M., Layman R., Chintalapani G. (2025). Gadolinium Nanoparticle-Enhanced, Radiopaque, and Resorbable Inferior Vena Cava Filters for Improved in Vivo Applications. Acta Biomater..

[87] Wang S.L., Siddiqui A., Rosenthal E. (2017). Long-Term Complications of Inferior Vena Cava Filters. J. Vasc. Surg. Venous Lymphat. Disord..

[88] Ayad M.T., Gillespie D.L. (2019). Long-Term Complications of Inferior Vena Cava Filters. J. Vasc. Surg. Venous Lymphat. Disord..

[89] PREPIC Study Group (2005). Eight-Year Follow-up of Patients with Permanent Vena Cava Filters in the Prevention of Pulmonary Embolism: The PREPIC (Prévention du Risque d’Embolie Pulmonaire par Interruption Cave) Randomized Study. Circulation.

[90] Soares J.S., Moore J.E. (2016). Biomechanical Challenges to Polymeric Biodegradable Stents. Ann. Biomed. Eng..

[91] Grogan J.A., O’Brien B.J., Leen S.B., McHugh P.E. (2011). A Corrosion Model for Bioabsorbable Metallic Stents. Acta Biomater..

[92] Dreher M.L., Nagaraja S., Batchelor B. (2016). Effects of Fatigue on the Chemical and Mechanical Degradation of Model Stent Sub-Units. J. Mech. Behav. Biomed. Mater..

[93] Mndlovu H., Kumar P., Du Toit L.C., Choonara Y.E. (2024). A Review of Biomaterial Degradation Assessment Approaches Employed in the Biomedical Field. npj Mater. Degrad..

[94] Ko K.-W., Choi B., Kang E.Y., Shin S.-W., Baek S.-W., Han D.K. (2021). The Antagonistic Effect of Magnesium Hydroxide Particles on Vascular Endothelial Activation Induced by Acidic PLGA Degradation Products. Biomater. Sci..

[95] Ma S., Feng X., Liu F., Wang B., Zhang H., Niu X. (2021). The Pro-Inflammatory Response of Macrophages Regulated by Acid Degradation Products of Poly(Lactide-Co-Glycolide) Nanoparticles. Eng. Life Sci..

[96] Shi W., Fuad A.R.M., Li Y., Wang Y., Huang J., Du R., Wang G., Wang Y., Yin T. (2023). Biodegradable Polymeric Nanoparticles Increase Risk of Cardiovascular Diseases by Inducing Endothelium Dysfunction and Inflammation. J. Nanobiotechnol..

[97] Wang X., Xiao Y., Wu B., Zhang X. (2011). Construction of Vena Cava Filter with Biodegradable Materials. Chin. J. Tissue Eng. Res. Clin. Rehabil..

[98] Xu W., Mou L., Mei J. (2023). Research Progress of Absorbable Inferior Vena Cava Filter. J. Vasc. Endovasc. Surg..

[99] Zhou B., Feng H., Wang Y., Wang X. (2020). Effect of different structural parameters of filter rod on mechanical properties of new vena cava filter. J. Biomed. Eng..

[100] Bajda J., Park A.N., Raj A., Raj R., Gorantla V.R. (2023). Inferior Vena Cava Filters and Complications: A Systematic Review. Cureus.

[101] Bikdeli B., Chatterjee S., Desai N.R., Kirtane A.J., Desai M.M., Bracken M.B., Spencer F.A., Monreal M., Goldhaber S.Z., Krumholz H.M. (2017). Inferior Vena Cava Filters to Prevent Pulmonary Embolism. J. Am. Coll. Cardiol..

